# Microwave synthesis of molybdenene from MoS_2_

**DOI:** 10.1038/s41565-023-01484-2

**Published:** 2023-09-04

**Authors:** Tumesh Kumar Sahu, Nishant Kumar, Sumit Chahal, Rajkumar Jana, Sumana Paul, Moumita Mukherjee, Amir H. Tavabi, Ayan Datta, Rafal E. Dunin-Borkowski, Ilia Valov, Alpana Nayak, Prashant Kumar

**Affiliations:** 1 Department of Physics, Indian Institute of Technology Patna, Bihar, India; 2 School of Chemical Sciences, Indian Association of Cultivation of Science, Kolkata, India; 3 Ernst Ruska-Centre for Microscopy and Spectroscopy with Electrons and Peter Grünberg Institute, Forschungszentrum Jülich, Jülich, Germany; 4 Peter Grünberg Institute (PGI-7), Forschungszentrum Jülich, Jülich, Germany; 5 Institute of Electrochemistry and Energy Systems, Bulgarian Academy of Sciences, Sofia, Bulgaria; 6 Global Innovative Centre for Advanced Nanomaterials, The University of Newcastle, Newcastle, New South Wales Australia; 7 Present Address: Department of Physics, Shri Ramdeobaba College of Engineering and Management, Nagpur, India

**Keywords:** Two-dimensional materials, Nanoscale materials

## Abstract

Dirac materials are characterized by the emergence of massless quasiparticles in their low-energy excitation spectrum that obey the Dirac Hamiltonian. Known examples of Dirac materials are topological insulators, *d*-wave superconductors, graphene, and Weyl and Dirac semimetals, representing a striking range of fundamental properties with potential disruptive applications. However, none of the Dirac materials identified so far shows metallic character. Here, we present evidence for the formation of free-standing molybdenene, a two-dimensional material composed of only Mo atoms. Using MoS_2_ as a precursor, we induced electric-field-assisted molybdenene growth under microwave irradiation. We observe the formation of millimetre-long whiskers following screw-dislocation growth, consisting of weakly bonded molybdenene sheets, which, upon exfoliation, show metallic character, with an electrical conductivity of ~940 S m^−1^. Molybdenene when hybridized with two-dimensional h-BN or MoS_2_, fetch tunable optical and electronic properties. As a proof of principle, we also demonstrate applications of molybdenene as a surface-enhanced Raman spectroscopy platform for molecular sensing, as a substrate for electron imaging and as a scanning probe microscope cantilever.

## Abstract (web summary)

A free-standing two-dimensional sheet composed solely of Mo atoms shows metallic character, with an electrical conductivity of ~940 S m^−1^.

## Main

Elemental Dirac and Dirac-like materials are of intense research interest, as they typically do not suffer from impurities in structural phases, offering enhanced electronic mobility, in contrast to other compound two-dimensional (2D) materials. Elemental Dirac materials include graphene^1^, borophene^2–4^, phosphorene^5^, silicene^6^, 2D gold^7^ and so on (Supplementary Fig. 1). Advancements in elemental Dirac materials are destined to involve higher-atomic-number metallic elements such as molybdenum, tungsten, titanium and so on, for which the sea of electrons would be confined in two dimensions, potentially leading to exotic electronic and excitonic behaviour. In addition, these materials are structurally robust under mechanical load and at elevated temperature^8^. Moreover, transition metals exhibit variable oxidation states, a property essential to catalyse chemical reactions, thus their 2D confined atomic sheets could demonstrate extremely high catalytic activity. The advantage of a 2D form of transition metals could also be exploited in niche areas, such as field emitters^9^, scanning tunnelling microscopy tips^10^, nanoscale interconnects^11^, nanoelectromechanical systems^12^ and surface-enhanced Raman spectroscopy (SERS)-based molecular sensing^13^. Growth of these 2D materials under ambient conditions is challenging due to their tendency to form clusters as well as their affinity towards oxygen^14^.

Here we provide evidence for the formation of free-standing atomic sheets of molybdenene, a 2D elemental Dirac material made of Mo atoms. We have prepared molybdenene under ambient conditions using microwave-induced electrochemical reduction of commercially available molybdenum disulfide (MoS_2_) powder. Molybdenene shows an electric conductivity of 940 S m^−1^. We demonstrate the use of molybdenene in atomic force microscopy (AFM) cantilevers, chemical and SERS-based molecular sensing and electron imaging. Ab initio density functional theory (DFT) study corroborates the experimental findings, predicting a stable staircase-like molybdenene sheet with fourfold symmetry, as well as van der Waals type interlayer interactions. We finally prepare 2D–2D hybrids of molybdenene with h-BN and MoS_2_. We have also shown the formation of molybdenene oxide.

## Experimental realization of molybdenene sheets

**Fig. 1:**
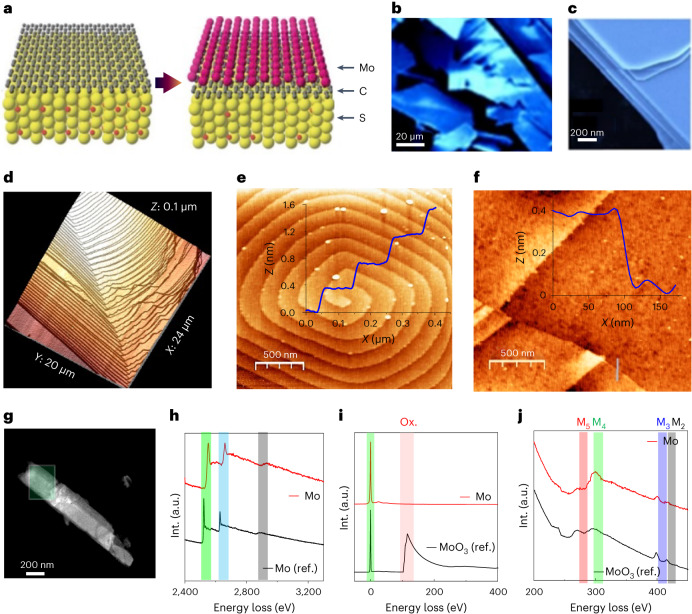
Experimental realization of molybdenene. **a**, Schematic diagram depicting graphene-catalysed microwave synthesis of molybdenene sheet (intense electric field breaks Mo–S bonds and Mo atoms move out through the expanded graphene network), Mo, S and C atoms are represented in pink, yellow and grey respectively, **b**,**c**, FESEM images of flat atomic sheets obtained by sonication followed by centrifugation of Mo whiskers. **d**,**e**, AFM images showcasing staircase-like features (each step being 0.4 nm) indicative of screw-dislocation-mediated growth of Mo whiskers (**d**,**e**) and showing monolayer Mo sheet, that is, molybdenene, transferred onto SiO_2_ substrate (**f**). **g**, Large-area HAADF image, which is taken to acquire EELS in the marked green region. **h**, EELS spectrum of synthesized Mo sheets in the high-loss region ranging from 2,400 to 3,300 eV. Int. intensity; a.u., arbitrary units. **i**, EELS spectrum ranging from 0 to 400 eV showing a zero-loss peak and low-loss plasmon oscillation peaks. **j**, EELS spectrum acquired in the high-loss region (200–450 eV) depicting a highly intense M_4_ peak.

Microwave-assisted field-induced electrochemical transformation of MoS_2_ results in the synthesis of molybdenene (schematic of synthesis in Fig. 1a, photograph of molybdenene whiskers in Supplementary Fig. 2 and details in Methods^15–17^). When observed under field emission scanning electron microscopy (FESEM), the whiskers clearly appeared to be a layered material with large surface areas (Fig. 1b,c; details in Supplementary Fig. 3). These whiskers, when exfoliated employing Scotch tape, resulted in atomically smooth metallic Mo atomic sheets, as observed in FESEM imaging. The formation of staircase-like features made up of atomic layers was revealed at the edges of the sheets, as observed in AFM images (Fig. 1d). On the surface, evidence of screw-dislocation-mediated staircase-like growth with step height of 0.4 nm, typical for single crystal monolayers, was observed (Fig. 1e). This growth mode has been already reported for GaN and MoS_2_^18–21^. A topography image of a molybdenene layer (thickness ~0.5 ± 0.05 nm), Scotch tape exfoliated and transferred to silicon substrate, is shown in Fig. 1f.

The electron energy-loss spectroscopy (EELS) spectrum of synthesized Mo sheets (large-area image in Fig. 1g) has two major high-loss peaks, L_3_ and L_2_, of Mo, at 2,552 and 2,660 eV respectively, and one minor edge peak, L_1_, at 2,924 eV, as shown in Fig. 1h**(**peaks left shifted due to weakening of interlayer coupling as compared with the bulk; https://eels.info/atlas/molybdenum). The low-loss region shows a zero-loss peak and low-loss plasmon oscillation peaks without any oxygen peak signals (Fig. 1i). The high-loss region (200–450 eV) beyond the low-loss plasmon region has two major edges of Mo, namely M_5_ and M_4_, which correspond to energy-loss near-edge structure, and two minor edges, M_3_ and M_2_, are extended energy-loss fine structure (Fig. 1j). Moreover, the highly intense M_4_ edge of synthesized Mo reflects the high concentration of the Mo atoms, which is expected in Mo in comparison with MoO_3_ (https://eels.info/atlas/molybdenum; ref. ^22^). Therefore, we can conclude that the synthesized 2D Mo is metallic in nature. Moreover, X-ray photoemission spectroscopy (XPS) study (Supplementary Fig. 4 and details in Supplementary Information) establishes that surface-bound oxygen can be easily etched out by Ar^+^ plasma. After 4 h of Ar^+^ plasma irradiation, we did indeed obtain XPS signal (Mo 3*d*_5/2_ peak at 228.13 eV; Supplementary Fig. 4e, f and Supplementary Table 1) from pure molybdenene metallic sheets. Supplementary Table 2, listing Mo 3d^5/2^ peak positions in XPS for pure molybdenum metal and its compounds, clearly indicates that we have obtained pure metallic material. Thus, EELS and XPS together prove the chemical phase purity of the synthesized molybdenene samples.

**Fig. 2:**
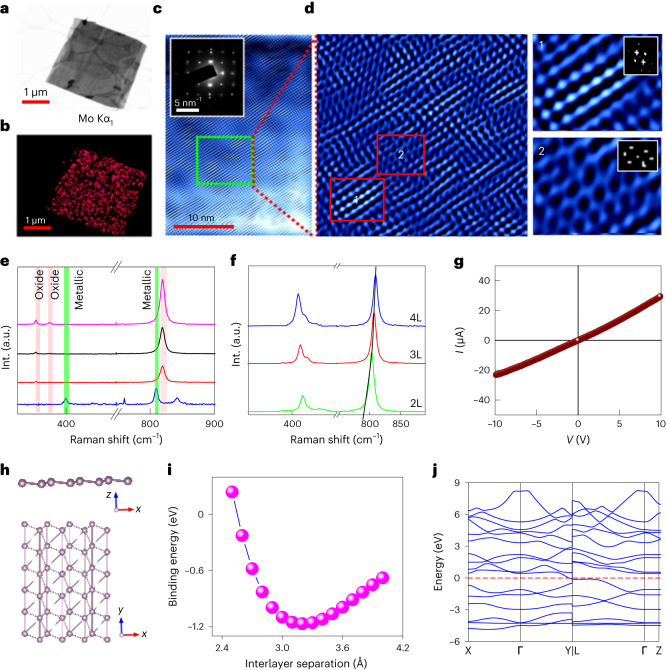
Structural details and electronic character. **a**,**b**, TEM imaging of formed molybdenene sheet and elemental mapping. **c**, HRTEM image of top surface of molybdenene. **d**, Zoomed-in image of marked region in **c**. Criss-cross patterns of atoms are observed with four-fold symmetry (region 1 in **d**) and hexagonal arrangements of atoms with sixfold symmetry (region 2 in **d**). **e**, Raman spectra of as-synthesized Mo whiskers and surface attained after subsequent peeling of top layer. The bottom spectrum for the final peeling step clearly shows distinct Raman peaks at ~405 cm^−1^, which is characteristic of metallic phase, and its second overtone at ~810 cm^−1^. For visual clarity oxide Raman peaks are red shaded and metallic Raman peaks are green shaded. Distinct metallic Raman signatures and flat sheets observed in FESEM, TEM and AFM establish synthesis of free-standing molybdenene. **f**, Layer dependence of molybdenene Raman spectra. **g**, *I*–*V* characteristics of Mo sheet placed on SiO_2_ substrate. **h**, Molybdenene structure with fourfold symmetry obtained using DFT calculations. **i**, The minimum-energy curve to determine equilibrium interlayer separation for molybdenene surface with fourfold symmetry. **j**, DFT band structure calculations of fourfold structure of molybdenene.

When scrutinized under high-resolution transmission electron microscopy (HRTEM), the molybdenene sheets were found to be entirely comprised of Mo atoms (Fig. 2a,b) and highly crystalline (Fig. 2c). Two distinct eigenstructures were revealed: one prominent phase with fourfold symmetry (region 1 in Fig. 2d and fast Fourier transform (FFT) in the inset) and the other with strain-mediated sixfold symmetry, found in very limited regions (region 2 in Fig. 2d and FFT in the inset). The areal number densities of atoms were 9.8 nm^−2^ and 8 nm^−2^ for the fourfold and sixfold symmetry phases respectively. Incidentally, our results for Mo sheets are very close to that for body-centred cubic single-crystalline molybdenum reported earlier^23,24^. Oxide formation can be safely excluded, as MoO_3_ and MoO_2_ exhibit higher interatomic distances ~3.6–4 Å and ~4.8–5.6 Å (refs. ^25–29^). Thus, the synthesized material is proved to be metallic structure-wise. In addition, we confirmed the single-crystalline nature of molybdenene sheets composed of whiskers with fourfold symmetry through the single-crystal X-ray diffraction pattern (Supplementary Fig. 5; details in Supplementary Information). The material was found to be triclinic with crystal parameters *a* = 3.70 Å, *b* = 3.97 Å, *c* = 13.90 Å, *α* = 89.75°, *β* = 89.97° and *γ* = 89.99°. It should be noted that the sheets exhibited ~3.5 times larger *c* parameter (characteristic of weak interlayer coupling) than the bulk Mo crystal, suggesting the arrangement of 2D sheets within the formed whisker.

Raman spectroscopy was also carried out to follow the exfoliation steps. As-prepared material exhibits Raman peaks at 284.01 cm^−1^, 364.5 cm^−1^ and 819 cm^−1^, which correspond to surface oxides (Fig. 2e). Upon successive exfoliations, the intensities of these peaks reduced. Moreover, after eight such top-layer removal exfoliation steps, Raman peaks arising due to pure metal (Mo–Mo) at 405 cm^−1^ and a second-harmonic peak at ~810 cm^−1^ were observed^30–32^. This establishes that oxygen is present primarily in the top few atomic layers of synthesized Mo material. However, the interior material just a few layers inside is still intact and metallic. This is quite natural, as the microwave exposure has been carried out in ambient conditions. Nevertheless, the energy-dispersive spectra of exfoliated oxygen-free molybdenene sheets, as shown in Supplementary Fig. 6, provided clear evidence for the high purity of these metallic sheets. Separately prepared Mo was also characterized by Raman spectroscopy, and it was established that characteristic Raman peaks corresponding to Mo appear (AFM images in Supplementary Fig. 7; Raman spectra in Supplementary Fig. 8). To establish the layer-number effect on the Raman peak positions, further exfoliation was carried out and it was found that the 810 cm^−1^ peak is shifted and is indeed sensitive to the number of layers (Fig. 2f), while on the other hand the 405 cm^−1^ peak does not shift much. Thinner sheets with fewer layers have less interlayer coupling; however, they couple better with silicon substrate. The metallic property of the molybdenene sheet was further confirmed by the Ohmic current–voltage (*I*–*V*) characteristics in two-probe *I*–*V* measurements using a Keithley instrument on molybdenene sheets on a lithographically fabricated device (Supplementary Fig. 9), as shown in Fig. 2g.

First-principles DFT study exhibits two constituent phases of molybdenene sheets, namely fourfold and sixfold symmetry phases (Fig. 2h and Supplementary Figs. 10 and 11) with Mo–Mo distance of ~2.9 Å (details in Supplementary Information). The fourfold symmetry phase consists of a staircase-like structure while the sixfold symmetry phase contains a graphene-like surface. The phonon dispersion spectra of the fourfold and sixfold molybdenene sheets exhibit dynamic as well as phase instability of the free-standing molybdenene sheets. However, the fourfold symmetry structure is more stable compared with the sixfold with relatively small imaginary frequencies (Supplementary Figs. 10 and 11; details of stability aspect in Supplementary Information). The interlayer interaction in molybdenene sheets can be considered as a van der Waals type interaction with an equilibrium interlayer separation 3.20 Å and binding energy −1.17 eV (Fig. 2i). However, the interlayer separation is not very different from the Mo–Mo in-plane bond distance (2.91 Å), which indicates that a very weak covalent interaction among the layers cannot be completely neglected. The metallic nature of atomic molybdenene sheets was further confirmed through DFT band structure calculations (Fig. 2j).

**Fig. 3:**
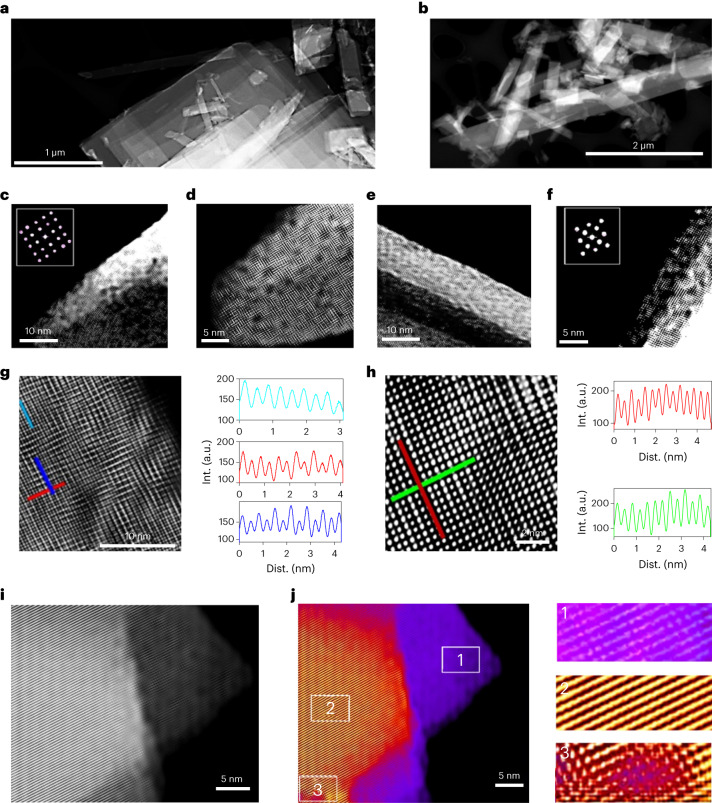
Atomic-resolution HAADF STEM imaging. **a**,**b**, Low-magnification HAADF STEM images of molybdenene highlighting the electron-transparent nature of the sheets. Stacked atomic layers are visible. **c**,**d**,**g**,**h**, High-resolution HAADF STEM images of different areas along with atomistic line profiles. Dist., distance. **e**,**f**, High-resolution cross-sectional images of molybdenene atomic sheets. Fourfold atomic arrangements are observed. **i**,**j**, Layer-dependent resolved images along with zoomed-in images in selected locations 1–3.

Thin metallic flakes were examined using high-angle annular dark-field (HAADF) scanning transmission electron microscopy (STEM) (Fig. 3a, b). Several rectangular atomic layers lying over others in a stacked manner are visible (Fig. 3a), consistent with the formation of molybdenene atomic sheets. The crystallographic structure evolution of molybdenene was explored by consecutive Ar plasma etching of the surface layers and subsequent HAADF STEM imaging. While our theoretical calculations confirmed the experimental result that the hexagonal phase is not stable and indeed was rarely observed, fourfold symmetry is a common feature in observation. Free-standing molybdenene sheets hanging over bare copper grids were imaged to find accurate structures (Fig. 3c,d,g,h). The theoretical interatomic distance of 0.32 nm indisputably validates the interatomic distance values of 0.33–0.36 nm 1.4 Å in graphene and 1.5 Å in borophene (due to the large atomic radius of 139 pm), experimentally observed broadly over synthesized molybdenene sheets at various locations and on different sheets, as evident from atomistic line profiles. It should be noted that the average interatomic distance in molybdenene is distinctly different from that in its 2D oxides (MoO_3_, ~3.6–4 Å; MoO_2_, ~4.8–5.6 Å). Cross-sectional images (Fig. 3e, f) further validate the square lattice appearing for sheets on copper support and hanging in empty space. The thickness variation in sheets that were hanging freely over empty spaces (Fig. 3i, j) could be resolved, and incidentally square lattices with interatomic distances of 0.33–0.36 nm were invariably observed for all numbers of layers down to monolayers.

Our theory suggests that molybdenene has staircase-like features, with alternate atomic arrays lying below and other atomic arrays lying above. In high-resolution HAADF STEM imaging, both arrays are visible. In some areas, we found linear arrangements of atoms (Fig. 3j2). Figure 3g,h shows intertwined atomic arrays. Line profiling vividly shows staircase and intertwined features. Free-standing molybdenene sheets exhibit local strain-mediated structural evolution in some areas (Fig. 3j3) (Supplementary Figs. 12–16; details of crystal growth aspect in Supplementary Information). Large-area HAADF STEM imaging (Supplementary Fig. 15) vividly exhibits a square lattice of molybdenene spread over the whole image area (~20 μm × 20 μm). Incidentally, the sheet area extends typically to 50 μm × 50 μm. Keeping in mind the challenges in growing Xenes such as the need for ultrahigh vacuum and expensive precursor gases for bottom-up atomic layer deposition/molecular beam epitaxy/chemical vapour deposition techniques and liquid phase exfoliation resulting in low-quality samples (lattice with structural defects and surface functionalities) with 50–500 nm lateral dimensions, the present report is certainly significant. In the present study, graphene acts as a crystallization catalyst, as it absorbs microwave energy and transfers this heat to MoS_2_ sheets adjacent to it. Local heat at the G–MoS_2_ interface results in electrochemical reaction, giving rise to decoupling of Mo atoms from S atoms, which makes clusters. We found moiré pattern features (Supplementary Fig. 17) in several areas during our detailed HAADF STEM imaging of molybdenene samples. This observation again attests to the formation of mono-/few-layered sheets. Thus, on the basis of various microscopies (FESEM, AFM, TEM and so on), especially HRTEM imaging and HAADF STEM high-resolution imaging, the atomic structure in molybdenene sheets was established. Raman spectroscopy, X-ray photoelectron spectroscopy and EELS established the chemical phase purity of the molybdenene sheets.

## Salient applications of molybdenene sheets

**Fig. 4:**
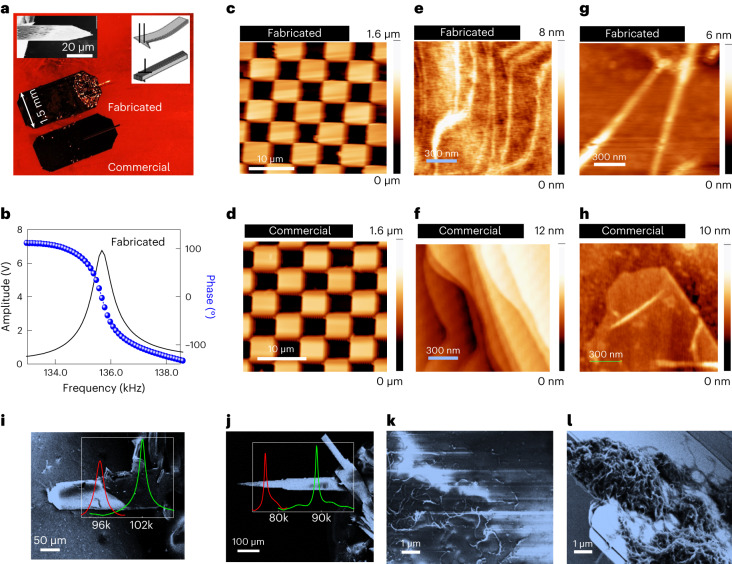
Applications of molybdenene. **a**, Optical images of fabricated and commercially available cantilevers. Top left inset: FESEM image of a typical molybdenene sheet used for cantilever fabrication. Top right inset: schematic depiction of parallel and perpendicular orientations of molybdenene sheets. **b**, Amplitude and phase versus frequency plots of a fabricated cantilever. **c**–**h**, Comparison of topography images of standard calibration grid (**c**,**d**), 2D structure of molybdenene sheets (**e**,**f**) and DNA on graphene oxide sheets (**g**,**h**) obtained with fabricated and commercial cantilevers, respectively. **i**,**j**, SEM images of Mo cantilevers (inset: amplitude (V) versus frequency (Hz) sweep before (green curve) and after (red curve) DNA attachment) for molybdenene-fabricated tips of two different dimensions (length and breadth). **k**,**l**, SEM images of multiwalled carbon nanotubes over glass and molybdenene as an anchoring substrate respectively.

Using molybdenene sheets of appropriate dimensions, we fabricated cantilevers for AFM by gluing them onto silicon chips (Fig. 4a). A typical molybdenene sheet chosen for cantilever fabrication is shown in the inset of Fig. 4a. The most salient feature of our cantilevers is that they are made up of layered material with in-plane covalent bonding and interplanar van der Waals interaction. This offers two possible orientations (inset of Fig. 4a). For the first orientation, where the layers are stacked parallel one above the other, the flexibility is dominant along the vertical direction and suppressed along the lateral direction^33^. This type of cantilever effectively minimizes the noise in the lateral direction, thereby making it suitable for high-resolution imaging in dynamic force mode. For the second orientation, where the layers are stacked laterally, the flexibility is limited in the vertical direction, thus minimizing the vertical deflection noise when used in lateral force mode^34^. We demonstrated successful imaging of versatile materials including hard silicon structures, smooth 2D surfaces and soft biomolecules using our cantilevers in the dynamic force mode. The tip was prepared by carefully bending the sharp end of the cantilever. The fabricated cantilever exhibited a resonance frequency of about 135 kHz and *Q* factor of about 160 (Fig. 4b). These values closely matched with those of the commercially available cantilevers. The topography images of a standard silicon calibration grating with a pitch of 10 µm (Fig. 4c,d), the surface of a molybdenene sheet with atomically resolved step heights (Fig. 4e,f) and DNA strands on a graphene oxide surface (Fig. 4g,h) obtained with the fabricated cantilevers were on a par with those of the commercial ones. It is noteworthy that our fabricated cantilevers are inherently metallic and highly reflecting, which enhances the deflection sensitivity of laser-based photodiode detectors. In addition, our cantilevers are advantageous over the commercially available metal-coated silicon cantilevers, which often fail due to delamination of the metal coating during *I*–*V* measurements/imaging. 2D materials have been employed as excellent nanomechanical resonators^35–37^ (details of resonator based on 2D materials and chemical sensing in Supplementary Information).

SEM images of fabricated Mo cantilevers are shown in Fig. 4i, j. The cantilever in Fig. 4i is shorter than that in Fig. 4j. The longer cantilever has lower fundamental resonant frequency (89 kHz) and the shorter has higher (102 kHz) (equation (1) of Supplementary Information). DNA detection has been carried out by attaching DNA to both the cantilever tips made up of Mo sheets; decrement in resonant frequency has been observed with the DNA attachment (equation (2) of Supplementary Information). In Fig. 4i the resonant frequency before DNA attachment was ~102 kHz (green curve) and it became 97 kHz (red curve) after DNA attachment. Similarly, in Fig. 4j (long cantilever), the resonant frequency before DNA attachment was ~89 kHz (green curve), and after DNA attachment it became 77 kHz (red curve). Secondary electrons are necessary in electron imaging through FESEM. Moreover, an atomically flat anchoring surface provides enhanced imaging. Molybdenene metallic 2D sheets were therefore explored as anchoring substrates for FESEM imaging. FESEM imaging of multiwalled carbon nanotubes over the glass substrate results in charging, which gives rise to poor contrast in images (Fig. 4k). However, charging is suppressed by taking molybdenene as an anchoring substrate for FESEM imaging of multiwalled carbon nanotubes and a clear topographic contrast is seen (Fig. 4l).

The surface morphology of molybdenene sheets revealed flat terraces and staircase structures with a significantly high step density. Low coordination number of edge atoms and high surface area-to-volume ratio make this material suitable for chemical sensing activities^38^. The chemical sensing properties have been tested by treating the material with ethanethiol, which showed substantial changes in molybdenene morphology due to successful molecular attachment. The AFM topography images of the surface of molybdenene sheets before and after the treatment with ethanethiol are shown in Supplementary Fig. 18. The surface stress and changes in mass and elasticity originating from molecular attachment play a crucial role in chemical sensing activity^39,40^. Hence, cantilevers made out of such material detect chemical species on the surface just by measuring the changes in frequency or force constant parameters. Molybdenene was also found to be competitive with gold and graphene^41^ as a material platform for SERS (Supplementary Fig. 19; details in Supplementary Information).

## Molybdenene-based 2D–2D hybrids

**Fig. 5:**
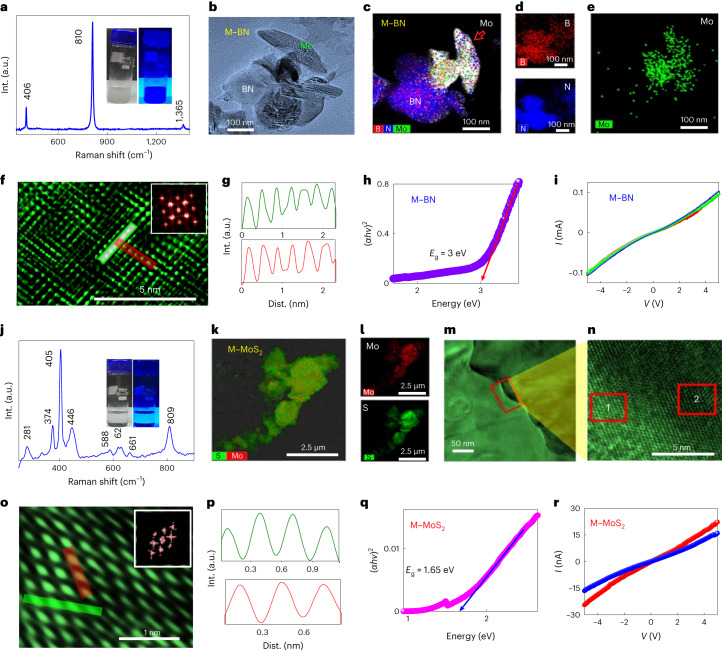
Molybdenene-based 2D–2D hybrids. **a**, Raman spectrum of synthesized M–BN hybrid and a digital image with UV light exposure (inset). **b**, TEM image of M–BN. **c**–**e**, Elemental mapping showing M–BN, boron, nitrogen and Mo. **f**, HRTEM image showing intertwined atomic arrangements. **g**, The atomic line profile of **f** as marked by the white and red lines has average interatomic distances of 0.35 nm and 0.35 nm. **h**, Tauc plot for optical band gap of M–BN hybrid. **i**, *I*–*V*/PC behaviour of M–BN hybrid. Red and green lines corresond to current signal under red or green laser illumination, respectively. **j**, Raman spectrum of synthesized M–MoS_2_ hybrid and a digital image with UV light exposure (inset). **k**,**l**, Elemental mapping of M–MoS_2_ hybrid, showing the presence of Mo and S. **m**, TEM image of M–MoS_2_. **n**, HRTEM image taken at the intersection of two layers. **o**, Zoomed-in HRTEM image of region 1 marked in **n** (inset: FFT pattern). **p**, The atomic line profile of **o** has average interatomic distances 0.31 (green plot) and 0.30 nm (red plot). **q**, Optical band gap plot of Mo–MoS_2_ hybrid. **r**, *I*–*V*/PC measurements of M–MoS_2_ hybrid. Blue and red lines corresond to current signal under blue or red laser illumination, respectively.

Raman spectroscopy of the M–BN hybrid shows peaks corresponding to both molybdenene (M) and BN (Fig. 5a). A photograph of the M–BN hybrid is shown in the inset of Fig. 5a. Structural modulation and reconfiguration of molybdenene is observed due to strong electrostatic interlayer coupling in these new van der Waals heterolayers. The TEM image along with elemental profiling of the molybdenene and BN hybrid (M–BN) (Fig. 5b–e) reveals overlying M and BN individual layers. The HRTEM image of the M–BN overlap area (Supplementary Fig. 20a) exhibits criss-cross patterns, where the inset selected area electron diffraction pattern depicts square crystal symmetry. HRTEM (region 1 marked in Supplementary Fig. 20a) indisputably reveals that an atomic sheet consisting of larger (Mo) atoms is positioned above another atomic sheet constituted of smaller atoms (B and N both being smaller). It should be noted that the role of the interlayer coupling between adjacent atomic sheets constituting 2D material hybrids in triggering atomic structure evolution/reconfigurations is crucial. The average interatomic distances of 0.35 nm were found to be equal along the two symmetry directions (Fig. 5f,g). The optical band gap (*E*_g_) determined from the Tauc plot is ~3 eV (Fig. 5h). Electrical and optoelectronic measurements confirm the semiconducting nature of the M–BN hybrid (Fig. 5i).

Raman spectroscopy of the M–MoS_2_ hybrid shows peaks corresponding to both MoS_2_ and molybdenene (Fig. 5j). A photograph of the M–MoS_2_ hybrid is shown in the inset of Fig. 5j. The TEM and elemental profile of the molybdenene and MoS_2_ hybrid (M–MoS_2_) exhibit two different statistically distributed structures with signatures of both M and MoS_2_ sheets (Fig. 5k–m). The HRTEM image of region 1 marked in Fig. 5n and the inset FFT pattern in Fig. 5o reveal stripe patterns of atoms in the M–MoS_2_ system with an average interatomic distance along two perpendicular directions of about 0.30 nm (Fig. 5p). In contrast, region 2 exhibits hexagonal symmetry in the crystal structure with average interatomic spacings of 0.16 nm and 0.32 nm respectively in the two directions (Supplementary Fig. 21a–c). The optical band gap was determined to be 1.65 eV (Fig. 5q). The semiconducting properties of M–MoS_2_ were confirmed by electrical measurement, and the optoelectronic characterization shows a red-sensitive photocurrent (PC) due to band edge absorption of the hybrid (Fig. 5r). Keeping the electronic character of 2D materials^42–46^ in perspective, molybdenene being metallic even in a monolayer, with sufficient carrier concentration and expected high mobility (in contrast to its bulk counterpart), thermal conductivity higher than in the bulk, enhanced flexibility and Young’s modulus higher than the bulk, will be apt for electrodes in electronics/optoelectronics, for energy storage and for catalysis. Molybdenene-based 2D–2D hybrids assume significance for coupling quantum states evolving at the interfaces playing a crucial role in determining physical/chemical properties of 2D–2D hybrids^47–49^.

## Conclusions

In summary, we have shown evidence for the formation of a new 2D Dirac elemental material we term molybdenene, through a facile single-step synthesis strategy via graphene-mediated microwave power absorption. Free-standing molybdenene sheets with atomically flat surfaces over large areas can be easily obtained by mechanical exfoliation of the initial molybdenene whiskers under ambient processing conditions within a few seconds. Additionally, 2D–2D hybrid materials with h-BN and MoS_2_ can be created with tunable structure and properties. We have demonstrated the successful application of molybdenene as scanning probe microscope cantilevers for both imaging and chemical detection and for chemical and SERS-based molecular sensing. Electron-rich molybdenene is suitable for imaging platforms and is expected to be an excellent catalyst in chemical reactions. The present study is poised to inspire molybdenene-based electronic devices and chemical sensors.

## Methods

### Experimental protocols

Initially MoS_2_ powder with 20 μm crystalline flakes was dried (at 150 °C on a hot plate for 2 h). To explore its role as a catalyst for the reaction, graphene powder was then mixed with the MoS_2_ (50:50 weight percentage). The mixed power was exposed to microwaves at 720 W in bursts of 10 s for up to 1 min in a closed alumina vessel under ambient conditions. After this process, the crucible was allowed to cool down for efficient heat dissipation to attain thermal equilibrium and avoid breaking the crucible as well as the bottom glass plate. The subsequent process was repeated three times to obtain sufficiently long whiskers. To avoid breaking the tip, we used electrostatic force to stick it on the sharp edge of the paper and gently place it in a collecting box. These microwhiskers were used for further characterization. The morphological features were studied using FESEM by transferring Mo needles on a conducting carbon tape. *I*–*V* measurements were made in a probe station at moderate vacuum conditions to avoid the oxidation of metallic needles. For photoconductivity measurements, blue, green and red lasers of wavelengths 505, 532 and 650 nm were used for optical excitation in PC measurements of Mo hybrids. Thiol functionalization was achieved by treating the obtained micrometre-sized needles in ethane dithiol solution for 24 h under ambient conditions. Treated samples were removed from solution and washed with deionized water to remove the unattached thiol group and dried under vacuum. A 10 ppm solution of methylene blue was used to demonstrate the SERS behaviour of Mo; we have also compared it with graphene and gold (gold sputtering done for 1 min on SiO_2_ substrate), which have good SERS capabilities.

### Synthesis of molybdenene and molybdenene oxide

MoS_2_ powder (purity 99.99%) and boron nitride powder (purity 99%) were purchased from Sigma Aldrich, graphene powder from Ultra Nanotech (99%), ethane dithiol (98%) from Merck and alumina substrate (99.7%) from Ants Ceramic. Isopropanol (IPA) was purchased from Merck (99%). All chemicals were used as received without any further purification. Molybdenene was synthesized by microwave exposure of a mixture of MoS_2_ and graphene powder in the ratio 1:1 under ambient conditions. MoS_2_ has lower thermal conductivity and higher dielectric loss than graphene, which supports heat accumulation at the interface. Graphene acts as a catalyst for effective absorption of microwave power, which is converted to heat and elevates the temperature. Consequently, microwave absorption further increases, which in turn further increases the temperature. This dynamic process is characterized by production of thermal spikes creating highly energetic, strongly nonlinear conditions at the interface. Details of microwave dissipation for 2D materials and its impact on peak temperature and rate of heating are presented in Supplementary Information.

While the generated thermal conditions instantly melt MoS_2_ (loosen bonds) as soon as the temperature reaches its melting point (*T*_m_ = 1,185 °C), the simultaneously acting local vertical electric field of the microwave being extremely high (>10^6^ V cm^−1^) results in breaking of Mo–S bonds. Released Mo atoms (rich in electrons) respond to such an enormous electric field and migrate through the graphene–MoS_2_ mixture, constituting a molybdenene layer. Anisotropy in growth features arises from the microwave electric field^16,17^. As time passes, several hotspots are generated and the whole surface is thermally activated, and enhanced participation of Mo atoms occurs, resulting in whisker formation. These whiskers consist of weakly bonded molybdenene sheets. The initial surface temperature increases with time as heat accumulates in the system, inducing a vertical thermal gradient. For this reason the bottom sheets grow larger than the topmost layer, giving rise to the staircase-like structure made up of molybdenene sheets. The process is schematically presented in Fig. 1a. As a result, we obtained molybdenene whiskers of variable length and width. They appeared highly reflecting and flat under an optical microscope with size ranging from a fraction of micrometre to a few millimetres. The essential role of graphene as a catalyst can be inferred from Supplementary Fig. 2. Molybdenene oxide (MO) was further produced from molybdenene by heating it at 200 °C and at 500 °C under ambient atmosphere for 2 h (AFM images in Supplementary Fig. 7 and Raman spectra in Supplementary Fig. 8).

### Synthesis of molybdenene-based 2D–2D hybrids

Hybrids of molybdenene with BN and MoS_2_ were synthesized sonochemically. First, the BN and MoS_2_ 2D materials were synthesized. To attain these, two different containers with dispersions of 50 mg of each of BN and MoS_2_ in 25 ml IPA were kept in an ultrasonicator (Cole Parmer, 40 kHz) and sonicated for 12 h. The supernatant was separated from each sonicated solution using a centrifuge (Remi, R-24) at a speed of 3,000 r.p.m., equivalent to a relative centrifugal force of 704 *g,* for 2 min. The obtained supernatant was dried at 100 °C for 2 h. For the M–BN synthesis, 5 mg of molybdenene was dispersed in 10 ml of IPA solvent along with 5 mg of synthesized 2D BN. The resultant mixture was then ultrasonicated for 6 h and thus M–BN hybrid was obtained. Similarly, for M–MoS_2_, 5 mg of each of molybdenene and synthesized MoS_2_ was dispersed in 10 ml of IPA solvent and sonicated for 6 h. The supernatants were taken from synthesized M–BN and M–MoS_2_ hybrid solutions and were used for further characterizations.

### Materials characterization

Synthesized 2D sheets of Mo were first diagnosed using an optical microscope (Olympus 100X). Single-crystal X-ray diffraction (AXS D8 Quest system from Bruker) was performed to check the type of crystal structure along with its related parameters. Further, its surface morphology was studied by FESEM (Hitachi S-4800) at operating voltage 30 kV and current 10 µA, placing the rods on carbon tape. TEM, HRTEM and selected area electron diffraction of free-standing Mo 2D sheets (dispersed in IPA sonicated for 4 h) were obtained using a JEOL JEM-2100. HAADF STEM images were recorded using a probe aberration-corrected FEI Titan G2 80-200 ChemiSTEM. AFM (Agilent model 5500) measurements were performed in non-contact mode to check minute details such as roughness and thickness of synthesized Mo sheets transferred to an Si/SiO_2_ substrate. XPS measurements of Mo sheets were performed in solid phase under high-vacuum conditions using an ESCA+ (Omicron NanoTechnology) to check their chemical purity. Raman spectroscopy of Mo sheets was carried out in solid phase before and after successive peeling steps within the wavenumber range of 100–1,500 cm^−1^ in the backscattering geometry employing a confocal micro-Raman spectrometer (Seki Technotron Corporation) with a 633 nm He–Ne laser source.

### Computation

All the spin-polarized calculations were performed within the framework of DFT using the plane-wave technique as implemented in the Vienna Ab Initio Simulation Package. The generalized gradient approximation method parameterized by the Perdew–Burke–Ernzerhof functional was used to account for the exchange–correlation energy^50^. The DFT + *U* method was used to account for the on-site Coulomb repulsion and improve the description of localized Mo *d* electrons in Mo sheet with *U*_eff_ = 2.0 eV as recommended by the previous studies^51,52^. The projector augmented wave potential was used to treat the ion–electron interactions. To describe the effect of van der Waals interactions, the DFT-D2 empirical correction method proposed by Grimme was applied^53^. In all computations, the kinetic energy cutoff is set to be 500 eV in the plane-wave expansion. All the structures were fully relaxed (both lattice constant and atomic position) using the conjugated gradient method, and the convergence threshold was set to be 10^−8^ eV in energy and 0.001 eV Å^−1^ in force. The Brillouin zone was sampled using a 9 × 9 × 1 Monkhorst–Pack *k*-point mesh for both geometry optimization and band structure calculation. Phonon dispersion spectra were obtained using the density functional perturbation theory as implemented in the PHONOPY code^54^. Ab initio molecular dynamics simulation was performed using the Vienna Ab Initio Simulation Package. Interlayer binding energy (*E*_b_) was calculated as *E*_b_ = *E*_bilayer_ − 2*E*_single layer_.

### Fabrication of AFM cantilever

Initially, the even-sized Mo whiskers were separated from the microwave-treated graphene–MoS_2_ powder mixture. Suitable Mo sheets were obtained by exfoliation. A commercially available silicon chip was used as a supporting material for the cantilever. Under an Olympus binocular microscope (CX-21), the chip and Mo sheets were arranged to fabricate a cantilever. Before fixing the rods on the chip, a small amount of Fevikwik instant glue was applied to give good adhesion. Following this protocol, more than ten cantilevers were prepared and tested in a Multimode Agilent 5500 AFM set-up. The AFM tip was prepared by gently bending the apex region of the cantilever using tweezers. The performance of each bent cantilever was then checked by scanning the standard calibration grids provided by Agilent AFM.

## Online content

Any methods, additional references, Nature Portfolio reporting summaries, source data, extended data, supplementary information, acknowledgements, peer review information; details of author contributions and competing interests; and statements of data and code availability are available at 10.1038/s41565-023-01484-2.

## Supplementary information

Supplementary Information Supplementary Figs. 1–21, Discussion (Sections 1–7) and Tables 1–4.

## Data Availability

Data are available upon request from the corresponding author.
