# Supplementary material for: Microwave synthesis of molybdenene from MoS2
Source: Nat Nanotechnol. 2023 Sep 4;18(12):1430–8. doi: 10.1038/s41565-023-01484-2 (PMC10716048; doi:10.1038/s41565-023-01484-2)
Supplement: Supplementary file 1 — Supplementary Figs. 1–21, Discussion (Sections 1–7) and Tables 1–4. [file 41565_2023_1484_MOESM1_ESM.pdf]

nature nanotechnology

Article

https://doi.org/10.1038/s41565-023-01484-2

Microwave synthesis of molybdenene from MoS_2

In the format provided by the
authors and unedited

Electronic Supplementary Information

Contents

Section 1. Microwave heating mechanism-**Page 2**
Section 2. X-ray photoelectron spectroscopy-**Page 5**
Section 3. Comparison of areal atomic number density of molybdenene with other 2D materials-**Page 9**
Section 4. Details of DFT theoretical calculation-**Page 14**
Section 5. Structural details from STEM HAADF imaging-**Page 18**
Section 6. Chemical sensing using AFM cantilever-**Page 22**
Section 7. SERS based molecular sensing-**Page 24**

Fig S1-Page 3
Fig S2-Page 4
Fig S3-Page 5
Fig S4-Page 7
Fig S5-Page 10
Fig S6-Page 11
Fig S7-Page 12
Fig S8-Page 13
Fig S9-Page 13
Fig S10-Page 14
Fig S11-Page 15
Fig S12-Page 16
Fig S13-Page 17
Fig S14-Page 19
Fig S15-Page 20
Fig S16-Page 21
Fig S17-Page 22
Fig S18-Page 23
Fig S19-Page 24
Fig S20-Page 25
Fig S21-Page 26

Table S1-Page 8
Table S2-Page 8
Table S3-Page 9
Table S4-Page 10

References-Page 27

1. Microwave heating mechanism

Microwave absorption power (**P_{abs}**) depends on frequency (**ω**) the dielectric constant (**ε**) of the material, electric field (**E**) component available in microwave and loss tangent (**tanδ**)[S1, S2] i.e.

$$\mathbf{P_{abs} = \frac{1}{2}\omega\varepsilon E^2 \tan\delta} \quad \text{.......................................[1]}$$

In the case of graphene, the direction of spontaneous electric field (primarily normal component) generated due to microwave exposure would determine local thermal conditions. The real part of the dielectric constant for graphene is high (~14) and would get enhanced at elevated thermal and field conditions. The dielectric constant of MoS_2 increases from ~5 to ~15 when the temperature is raised from 200 to 800 °C for 2.22 GHz[S3]. Incidentally, relaxation time for electrons i.e. Dirac massless fermions, in graphene is 250-450 fs[S4,S5]. Earlier, graphene, MoS_2, and MoS_2-Graphene have been told to be microwave absorbers[S12,S13]. Interfaces of layered materials in general exhibit exceptionally high dielectric function, due to Maxwell–Wagner polarization effect[S6]. The estimated local temperatures under microwave (2.45 GHz, 720 W) exposure for ~2 minutes using Equation (1) are ~1800 ºC for MoS_2 only and ~3000 ºC for MoS_2+G mixture, which exceeds the melting point of MoS_2[S7].

In fact, heating rate **δT/δt** under microwave exposure is given by[S2]

$$\frac{\delta T}{\delta t} = \frac{K\varepsilon'' f E^2}{\rho C_p} \quad \text{...[2]}$$

Where $\boldsymbol{K}$ is proportionality constant, ε'' is imaginary part of the dielectric constant of the material, f is the frequency of the electromagnetic wave, $\boldsymbol{E}$ is the electric field intensity, $\boldsymbol{\rho}$ is the density of material and $\boldsymbol{C_p}$ is the specific heat of the material. For graphene, $\boldsymbol{\varepsilon''}$ is ~14, $\boldsymbol{\rho}$ is ~ 2.27 g/cm^3 and $\boldsymbol{C_p}$ is ~5 J/mol.K. Similarly, for MoS_2 $\boldsymbol{\varepsilon''}$ is ~16.3, $\boldsymbol{\rho}$ is 5.06 g/cm^3 and $\boldsymbol{C_p}$ is ~ 62.23 J/mol K[S8-S11]. Using Equation (2), the rate of heating for 20 s microwave pulses for G has been estimated in the present case to be 2.66 times higher than that of MoS_2.

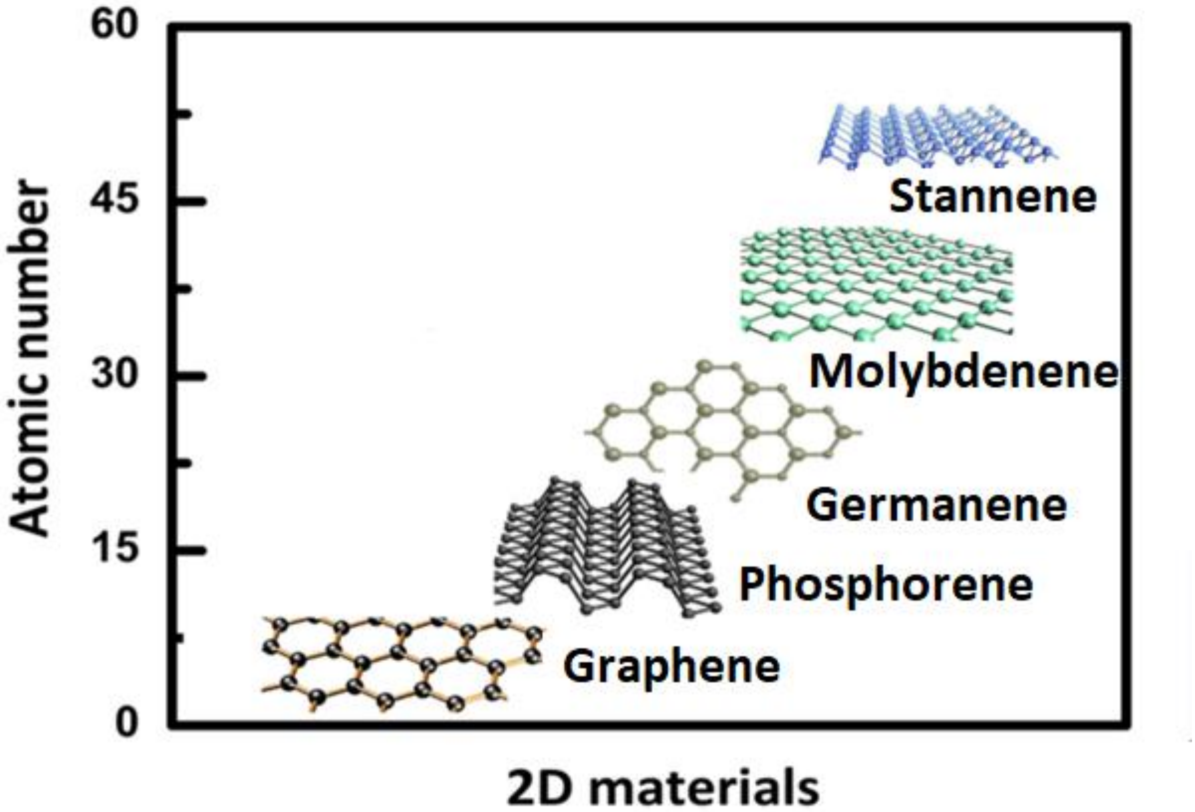

Fig S1 Panoramic view of evolution of Xenes (family of elemental atomic sheets). Energetically stable structures are shown for representation. However, there are several structural phases of each of these family members.

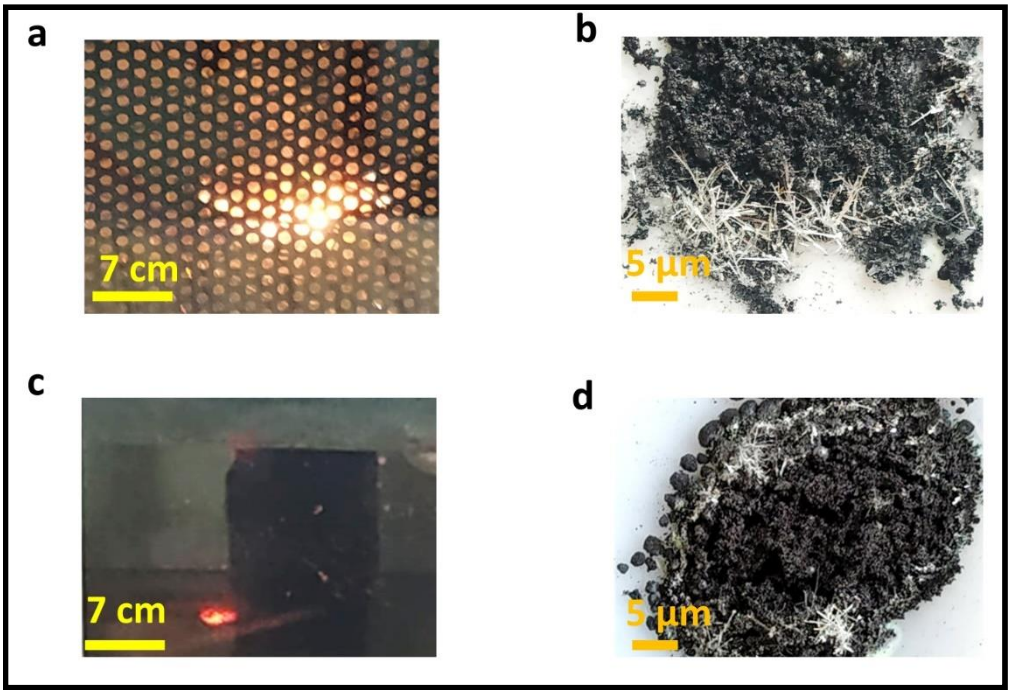

Fig S2 Camera photograph of (a) generated plasma and (b) formed Mo whiskers when powder mixture of MoS_2 and graphene was exposed to microwave for ~1 min. Corresponding photographs of (c) plasma and (d) Mo whiskers when MoS_2 powder was microwave exposed without graphene.

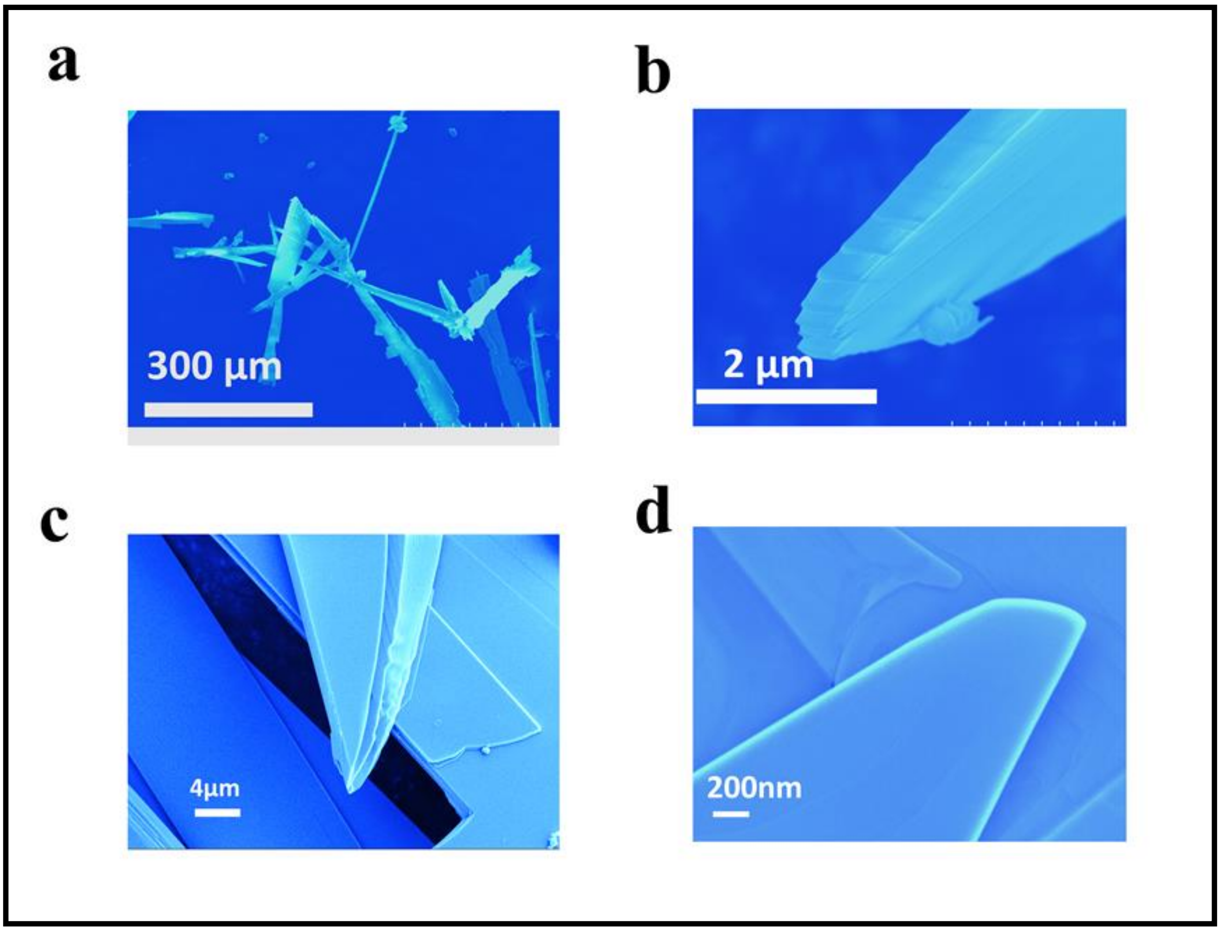

Fig S3 (a) Large area FESEM image of synthesized Mo whiskers. Closer look reveals sheets with sharp tips, sheet-folds and their joints. (b) Side view image of one such whiskers showing layered structure. (c) Large area and (d) zoomed-in FESEM image for micromechanically transferred Mo sheet on SiO_2 substrate. Imaging was carried out without any gold coating, yet no charging was evidenced revealing their electronically conducting nature.

2. X-ray photoelectron spectroscopy

The XPS of as synthesized molybdenene exhibits the 3d5/2 and 3d3/2 peaks at 232.09 eV and 235.23 eV respectively (**Fig S4 (a-b), Table S1**), which indicates the oxidation of the molybdenene surface. In molybdenene oxides, the molybdenum atom is bonded to oxygen atoms, which results in a redistribution of the electron density around the atom. This leads to a reduction in the screening of the core electrons, which in turn increases their binding energy. The Mo-3d peak will typically be shifted to higher binding energies, reflecting the more electronegative chemical environment in the oxide compared to the metal. The XPS spectrum of surface oxidized molybdenene typically shows a broader and more complex Mo 3d peak

than that of metallic molybdenene, which is due to the presence of different oxidation states of molybdenum in the oxide. The Mo 3d peak is split into multiple components, with each component corresponding to a different oxidation state[S14-15]. When we compared our results with the literature (**Table S2**), we find that the XPS peaks indicates the formation of molybdenene oxide between MoO_2 and MoO_3. After 2 hr of Argon plasma cleaning, Mo-3d5/2 and Mo-3d3/2 peaks shift to 229.16 eV and 232.46 eV (**Fig S4 (c-d), Table S1**), which hints at the presence of MoO_2 as evident from **Table S2**. To get even better signal from the underlying molybdenene, we prepared another sample with 4 hours of continuous Ar plasma etching and interestingly we got Mo-3d5/2 and Mo-3d3/2 peaks 228.13 and 231.28 eV (**Fig S4 (e-f), Table S1**), which corresponds to metallic molybdenum (**Table S2**). It should be noted that according to most of the literature, bulk metallic Mo exhibits $3d^{5/2}$ peak at 227.91 eV.[S16] In general, inter-layer coupling weakens when one goes from the bulk to few monolayers. Due to weakening of inter-layer coupling, in-plane binding energy should naturally increase. For example, lower number of layers of germanene has been reported to have higher binding energy[S17]. However, it should be kept in mind that layer-dependent shift in binding energy is usually ~ 0.2 eV.

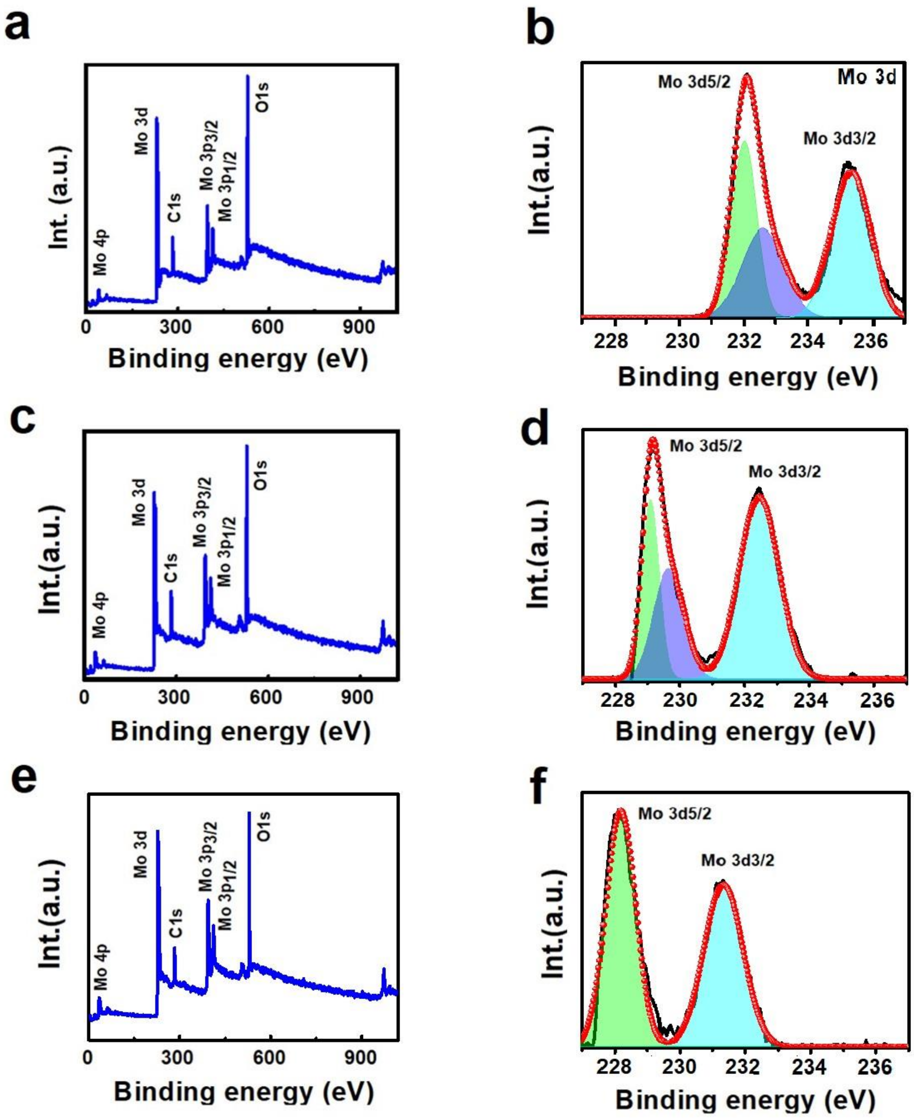

Fig S4. XPS spectra (survey and 3d resolved peaks) for molybdenene samples for (a,b) as synthesized, (c,d) after 2 hours of Ar^+ plasma etching and (e,f) after 4 hours of Ar^+ plasma etching.

	Mo	Mo (2 hr.)	Mo (4 hrs.)
**B.E. (eV) Mo $3d_{5/2}$**	**232.09**	**229.16**	**228.13**
**B.E. (eV) Mo $3d_{3/2}$**	**235.23**	**232.46**	**231.28**

Table S1. XPS peak positions for $3d_{5/2}$ and $3d_{3/2}$ for as synthesized molybdenene sample and for samples obtained after Ar plasma irradiation for 2 and 4 hours.

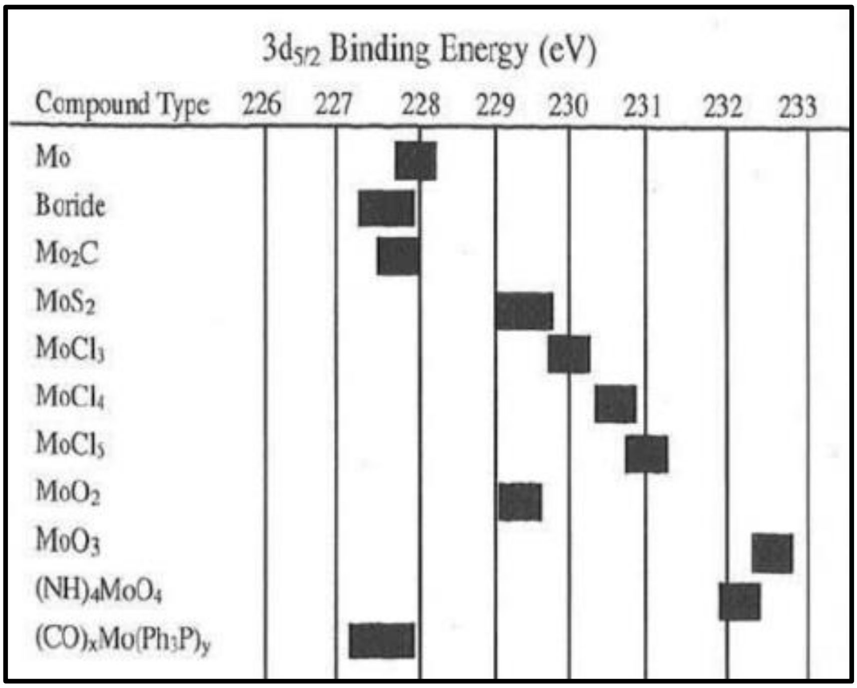

Table S2: Mo $3d^{5/2}$ peak position for pure metallic and various molybdenum compounds[S18]

Materials	Interatomic distances	
MoO_3	3.6-4 Å	
MoO_2	4.8-5.6 Å	
Mo	2.5-2.8 Å	3.1-3.3 Å

Table S3: Average inter-atomic distances observed in HRTEM of molybdenene in present case (Mo) *vis-a-vis* those for its two oxides namely MoO_2 and MoO_3. Distance between Mo atoms in molybdenene in the present research is ~3 Å. Incidentally, inter-atomic distance observed for Mo sheet in our investigation is the least.

3. Comparison of areal atomic number density of molybdenene with other 2D materials

As areal number density of atoms is considered to be an excellent and essential parameter to distinguish 2D phases from its bulk, this criterion was also critically discussed for M sheet and the result was compared with other 2D materials e.g., graphene, borophene, phosphorene, silicene. Most of the light elemental flat atomic sheets have high atomic packing density and hence high atomic number density ~30-40 nm^{-2}, except for phosphorene and borophene (β_{12}) which have 3D protrusion embedded in their atomic structures. In contrast Mo atoms possess large number of electrons with respect to lighter atoms and of this reason, Molybdenene sheet exhibited atomic number density ~8-9 nm^{-2}, keeping large distance between Mo atoms in the 2D lattice (Table S4). This value of areal density arises as Mo atoms are very large in dimension and therefore huge number of electrons (42) compared to low atomic number Xenes such as graphene (6), borophene (5) and even phosphorene (15). Electron clouds in one atom repel those from the other atoms and electron-nuclei attraction balances and an equilibrium distance is therefore maintained between the atoms.

Atomic profiles generated from HRTEM images were minutely analyzed and we found that Mo-Mo inter-atomic distances are 2.5-2.8 Å and 3.1-3.3 Å in directions normal to each other

(Table S3, details in ESI). Due to high atomic number, molybdenum atoms keep large distance apart from each other and hence small areal number density.

Materials	Atomic number density (nm^{-2})
Graphene	~37-38
Phosphorene	~15-16
Silicene	~38
Borophene	~31-33
Molybdenene	~8-9

Table S4: Atomic number densities observed for molybdenene sheet along with other elemental 2D materials (Xenes).

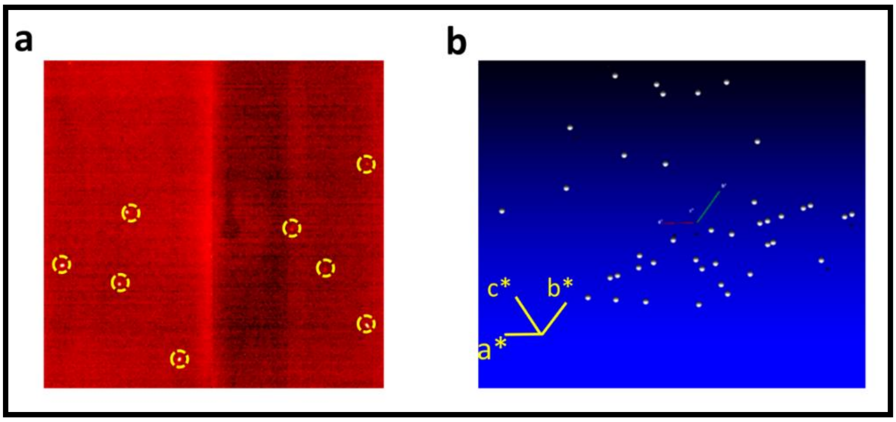

Fig S5 (a) Single crystal XRD pattern obtained for Mo whiskers using BRUKER AXS D8 Quest System. Bright spots (highlighted by yellow circles enclosing them) correspond to family of planes and (b) atomic arrangements in Fourier space derived from the measurement. Crystal structure obtained was triclinic with crystal parameters a=3.70 Å, b = 3.97 Å, c = 13.90 Å, α=89.75°, β=89.97° and γ= 89.99°. It should be noted here that c-parameter is about 3.5 times higher than the bulk 3D molybdenum metal.

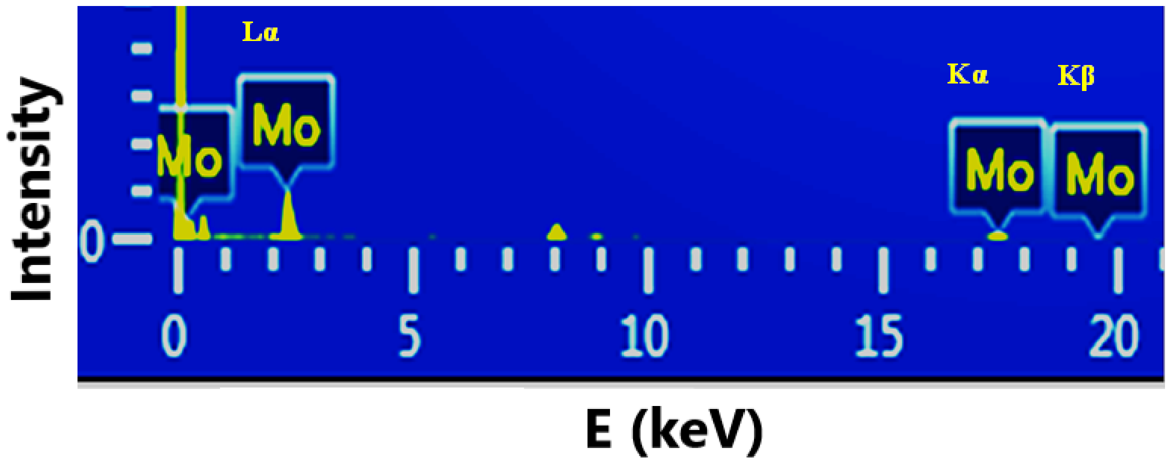

Fig S6 Energy dispersive spectra (EDS) acquired for synthesized Mo sheets. This result proves structural purity.

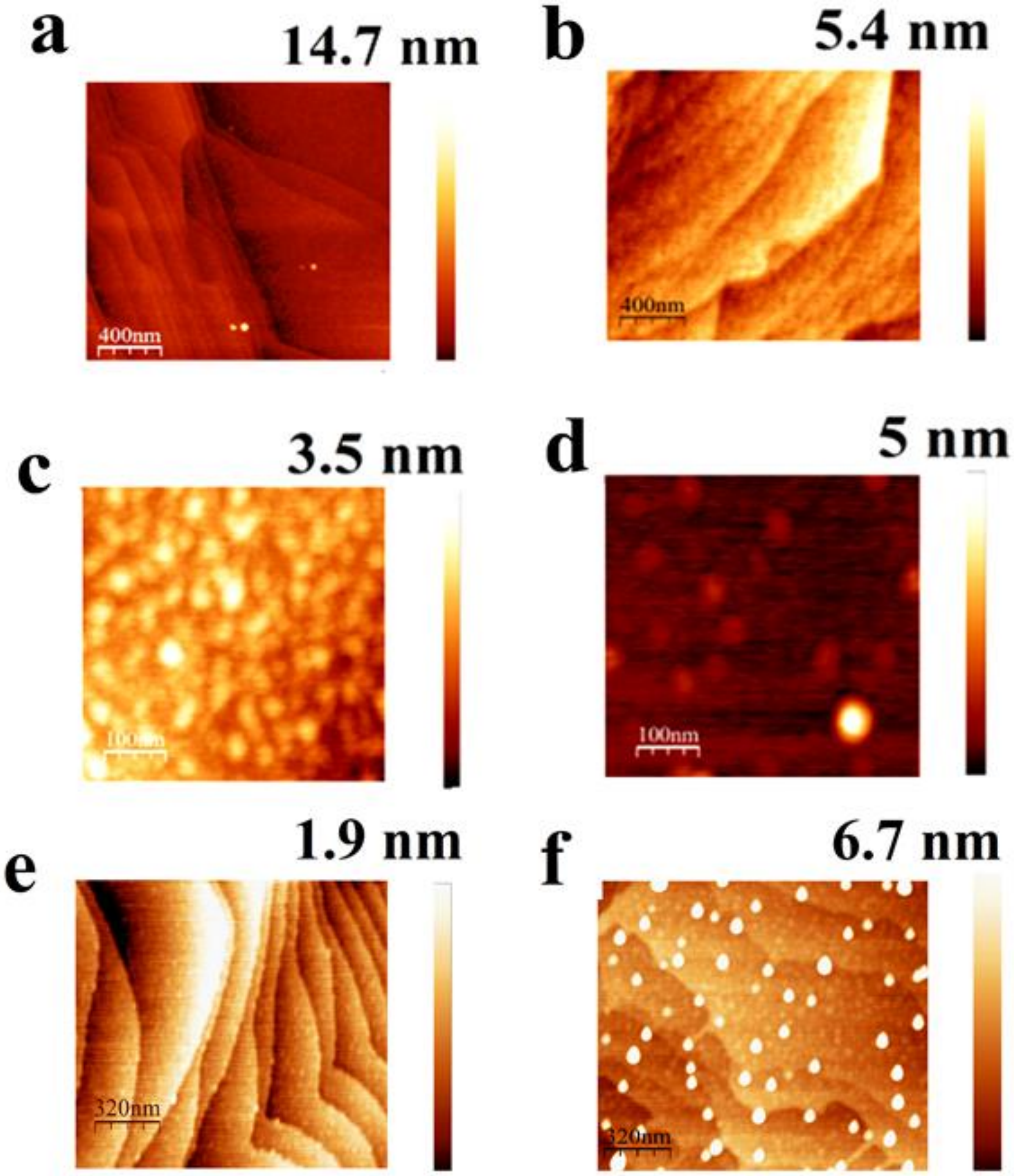

Fig S7 AFM image of (a) freshly prepared sample (very smooth surface with no blister) and (b) sample placed at moderate vacuum (10^{-3} mbar) inside a desiccator for 10 days (no significant changes observed), (c) sample intentionally left in humid environments for 10 days (whole surface area covered with small blisters confirming high oxidation of metallic sheets), (d) sample surface after micromechanical peeling of top layers using scotch tape (significant reduction of blisters), (e) sample after intentional heating at 200 °C for 2 hrs (small blister formation initiated), (f) sample after heating (200 °C for 2 hrs) under humid environment (dense blisters form with significant heights).

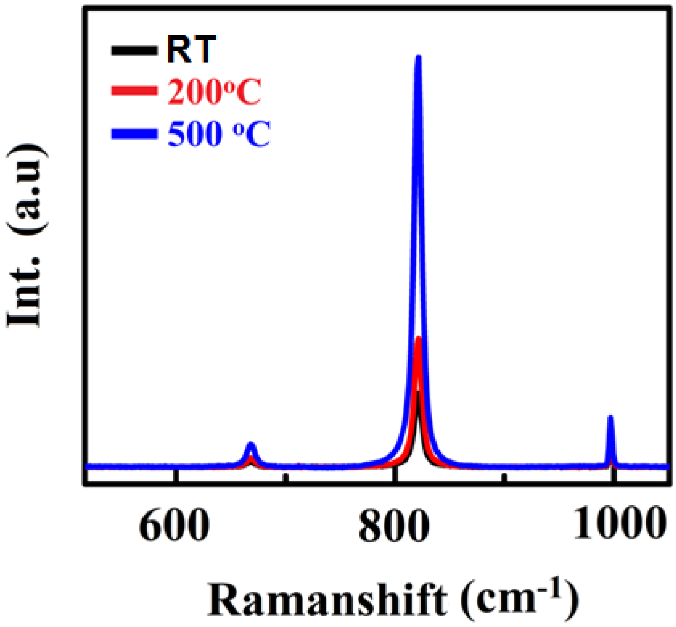

Fig S8 Raman spectra of Mo sheets for as synthesized sample and samples obtained after heating at 200 and 500 °C for 2 hr.

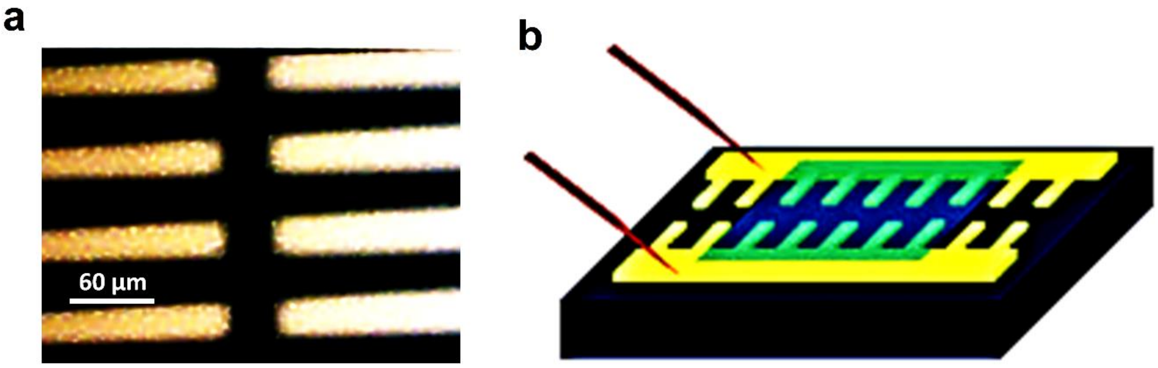

Fig S9. Two probe electrical measurements with lithographically fabricated electrodes.

4. Details of DFT theoretical calculation

Phonon mode analysis reveals that this small instability in M sheet with four-fold symmetry arises due to positional instability of the Mo atoms along y and z-direction. The instability occurs due to presence of high strain in M sheets. Moreover, the root-mean-square deviation (RMSD) as well as radial distribution function plots (obtained from AIMD study) demonstrates high thermal stability of four-fold symmetry M sheet while six-fold M sheet with hexagonal arrangement exhibits instability under ambient condition (Figures ESI S10-S11).

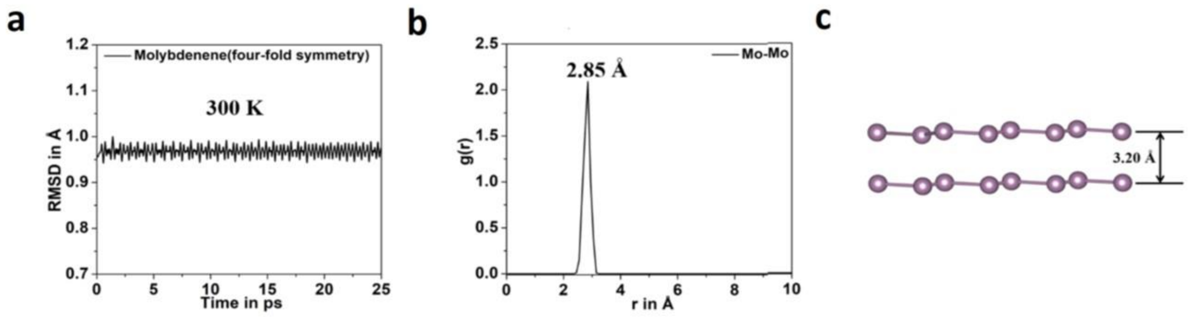

Fig S10 (a) Plot of RMSD vs time at 300 K. (b) Plot of radial distribution function {g(r)} between Mo-Mo pair. (c) structure of molybdenene bilayer with equilibrium interlayer separation.

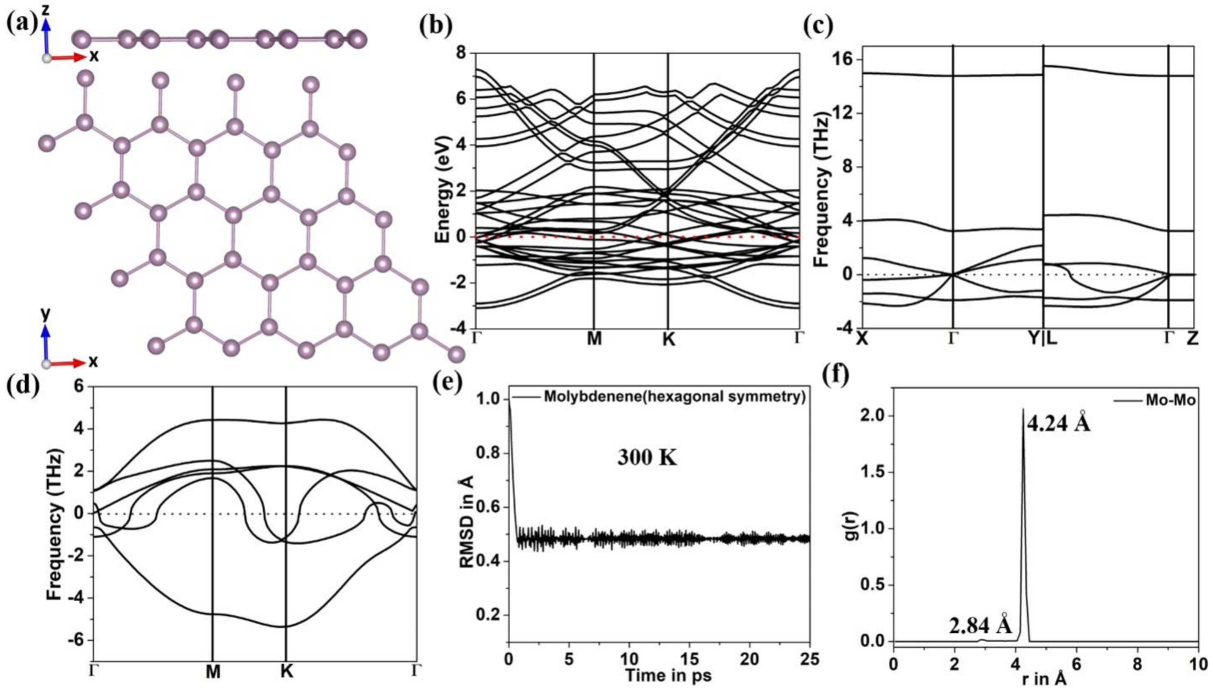

Fig S11 (a) Molybdenene surface with six-fold symmetry obtained by DFT calculations. (b) DFT band structure. Phonon dispersion spectra (c) four-fold symmetry, (d) six-fold symmetry surfaces. (e) Plot of RMSD vs time at 300 K, (f) Plot of radial distribution function {g(r)} between Mo-Mo pair for molybdenene surface with six-fold symmetry.

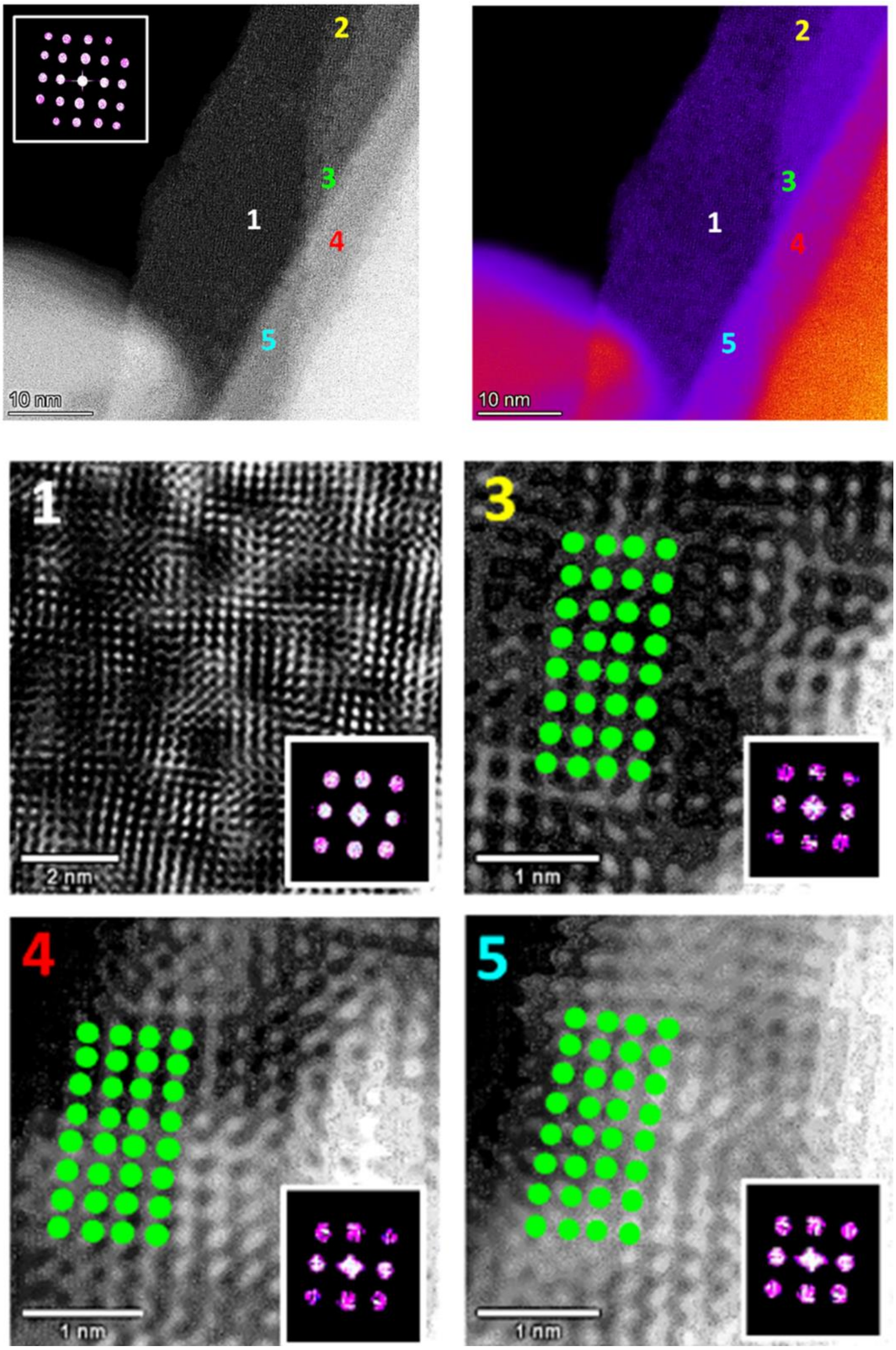

Fig S12 Revelations of layer dependent structure by STEM HAADF.

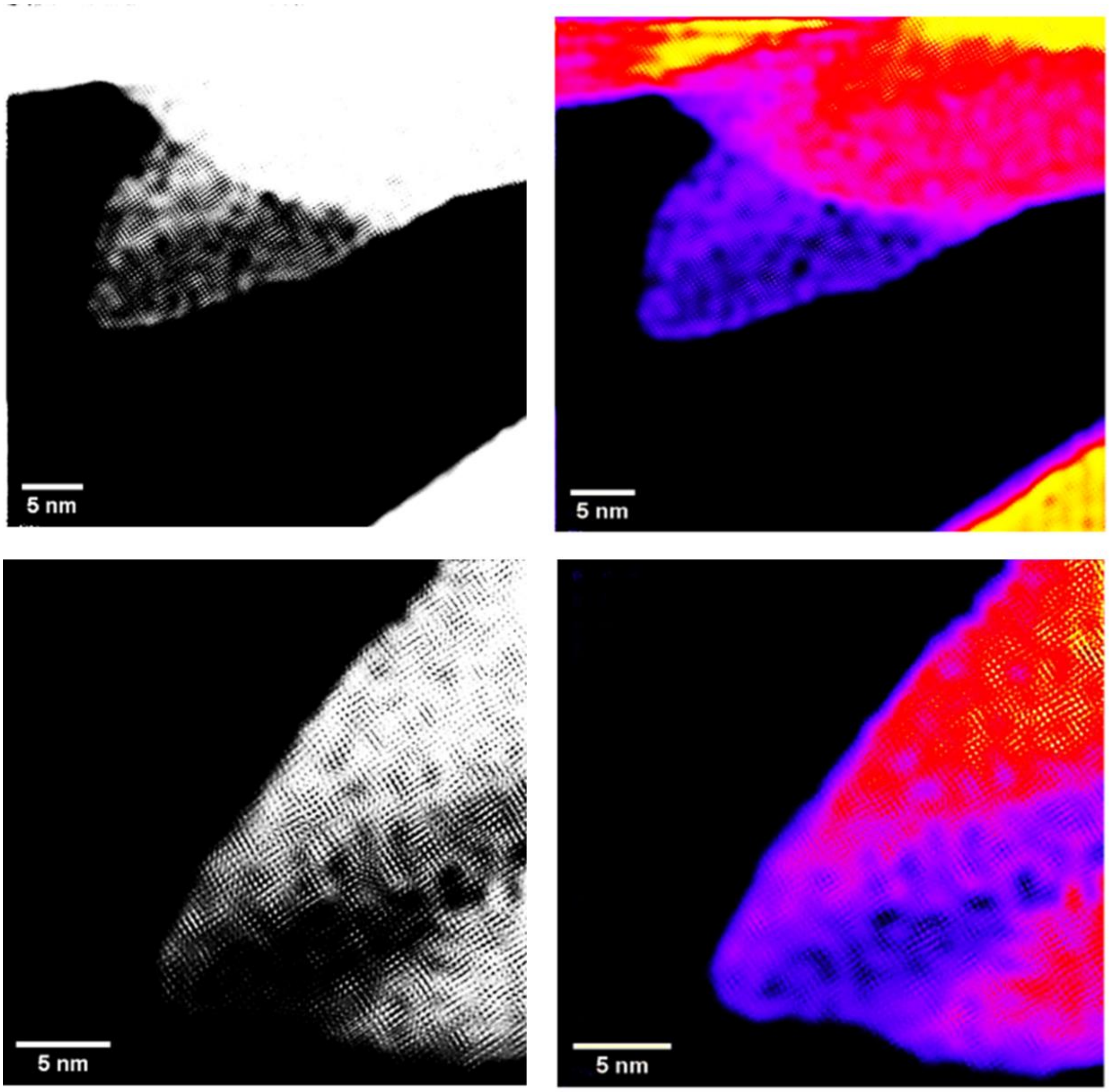

Fig S13 Revelations of layer dependent structure by STEM HAADF.

5. Structural details from STEM HAADF imaging:

Even though graphene, MoS_2, and molybdenene were present in the reaction product, isolation of molybdenene whiskers was very easy from the reaction product. We could also observe features at the interface with graphene as well as with MoS_2, where crystal lattice features with high strains were apparent (Fig S14). Inter-atomic distance in those locations were found to be 2-2.5 Å. In some locations (Fig S14 (c)), atoms are very closely spaced in atomic chains, which are smaller too, those underlying atoms most possibly are carbon atoms of graphene catalyst. For the first layer of growth of molybdenene on graphene, strain will have a significant effect and the presence of in-plane four-fold symmetry with the ideal lattice parameter of molybdenene is difficult due to high residual strain in the system. In general, local defects formation relieve local strain (for example structure in Fig S14). Especially as it reports on new metallic Xene. Reports of lateral dimensions ~5-20 μm are rare though. Observation of strain in molybdenene sheets have been made in some area. For example, see large area image HAADF STEM image (Fig S16) of sheets with local strain and intertwined structural features. Bulk material usually does not exhibit such out-of-plane protruded features. These features are characteristics of mono/few layers of 2D materials. Moreover, Moire patterns forms in 2D materials when two successive layers are at some angles, or displacement, or both.

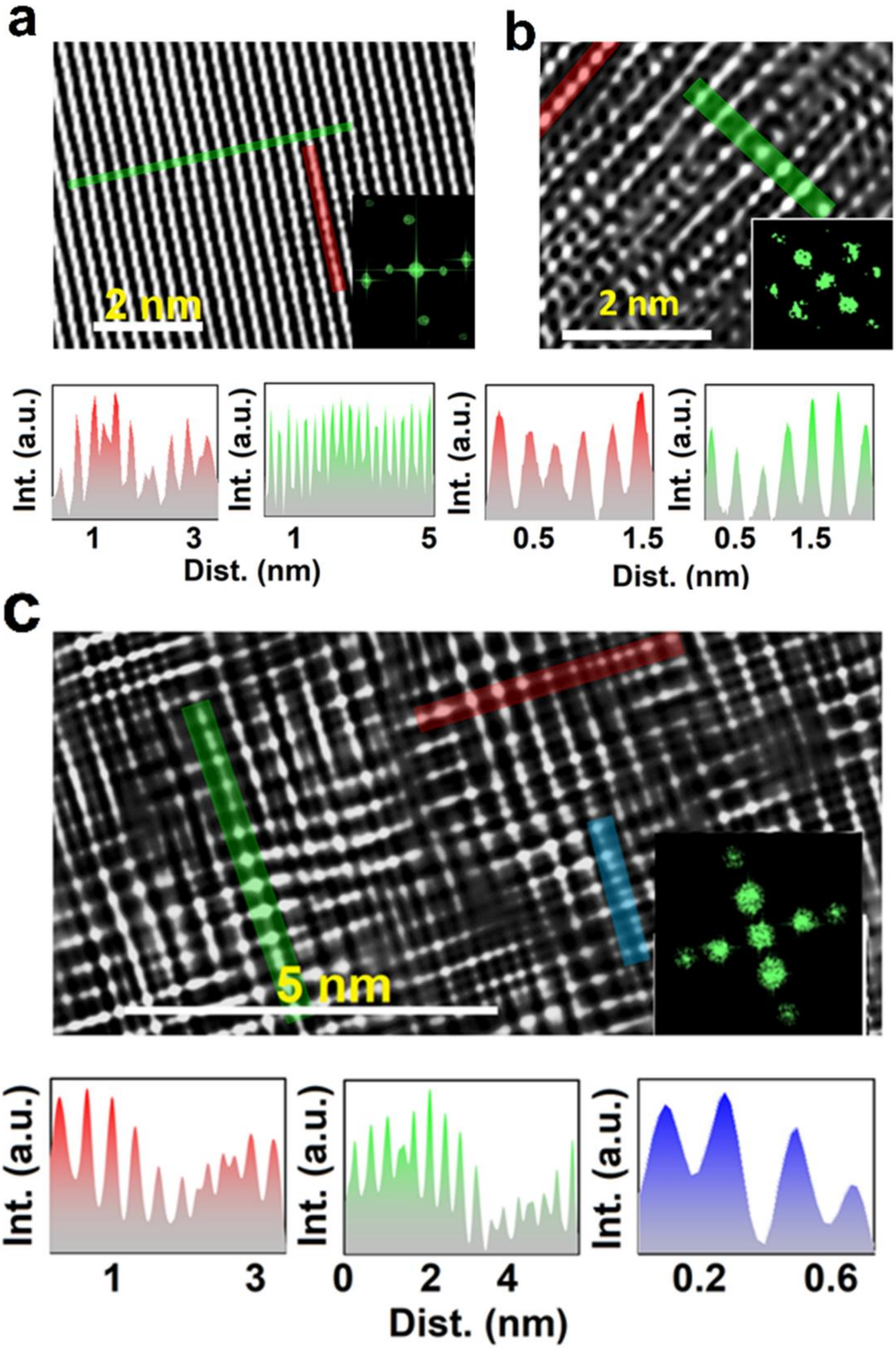

Fig S14 Revelations of strained interfaces by STEM HAADF.

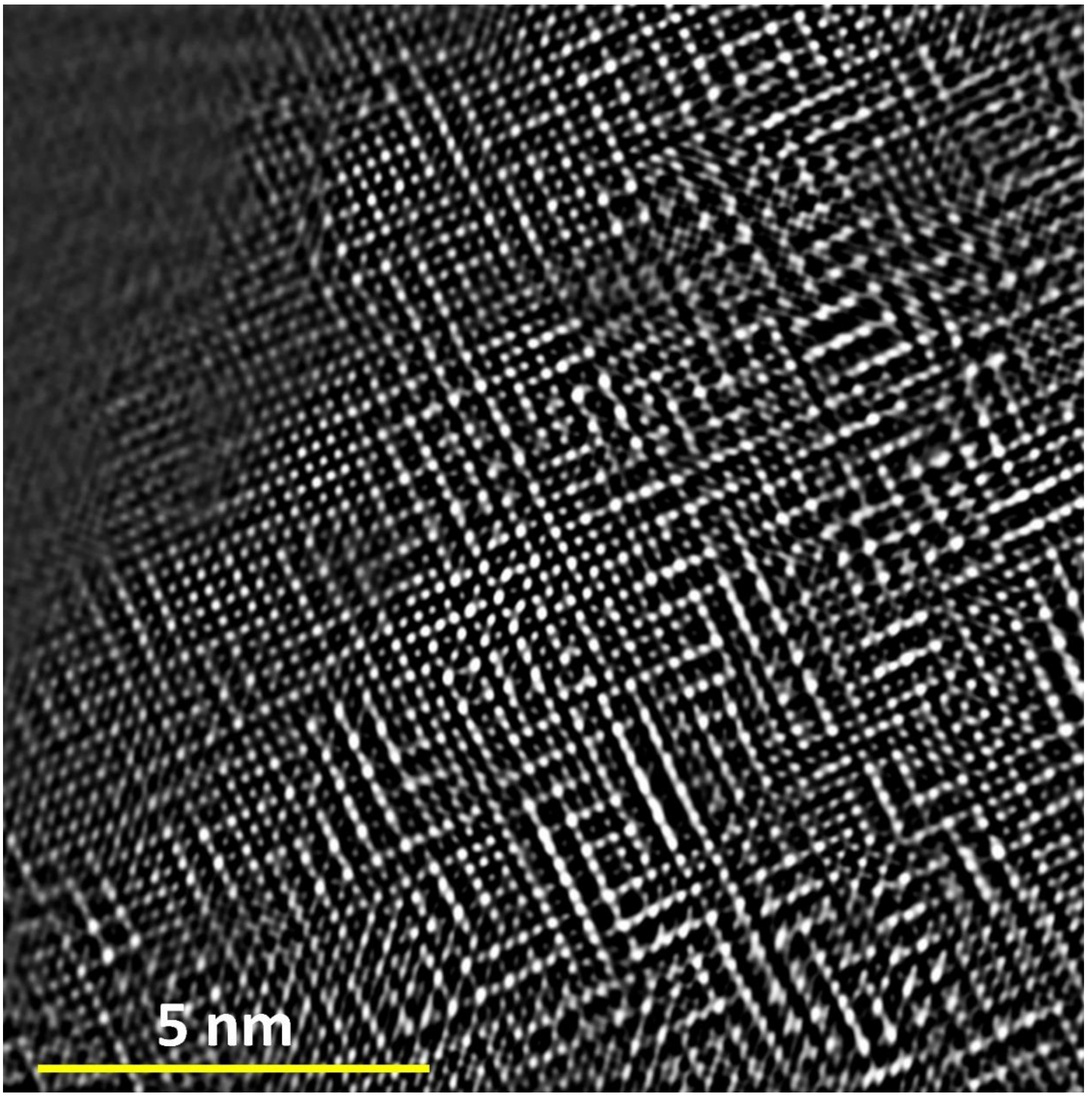

Fig S15. Large area STEM HAADF image showing square lattice symmetry.

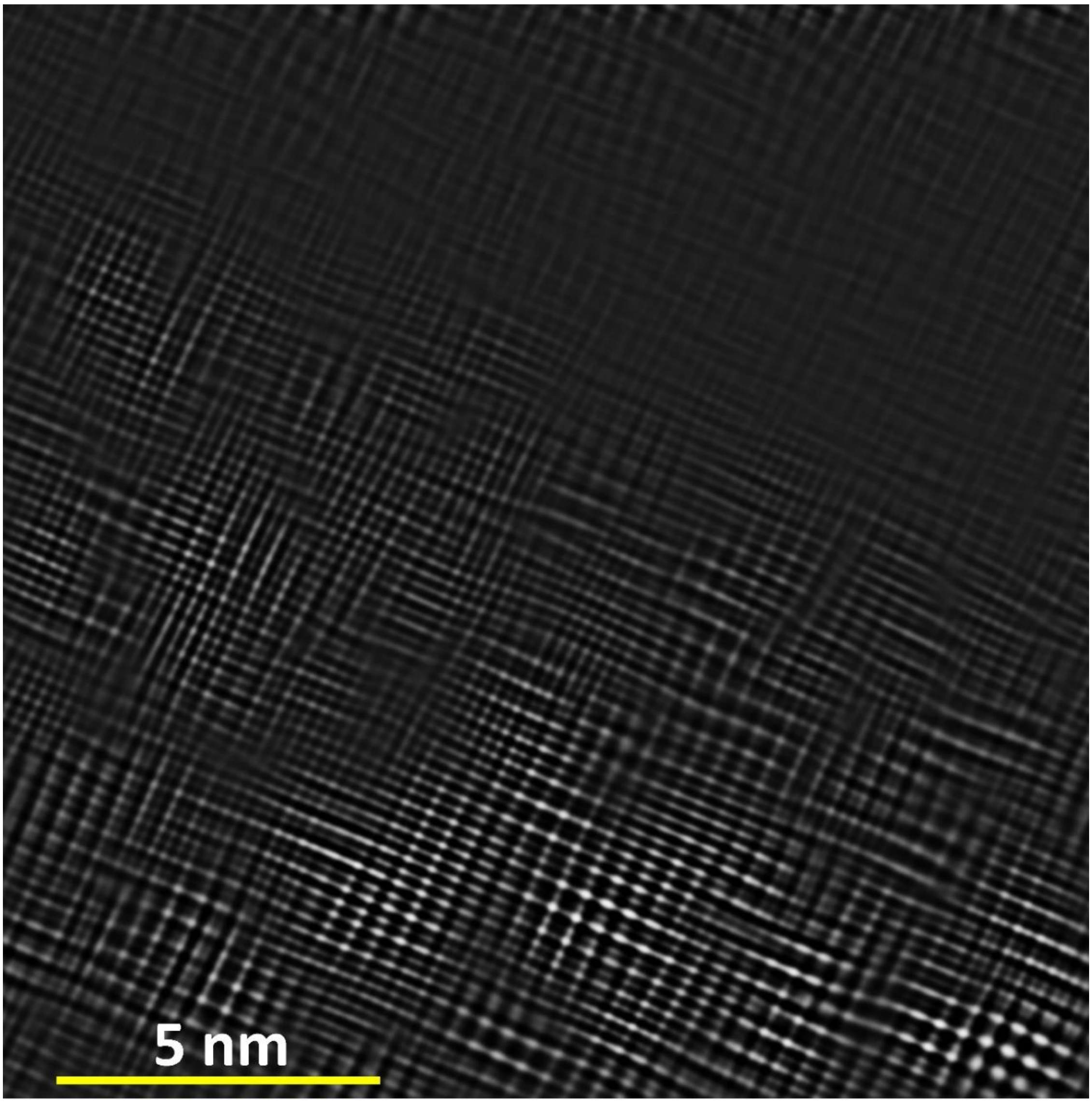

Fig S16. Large area STEM HAADF image showing local-strained (intertwined) features.

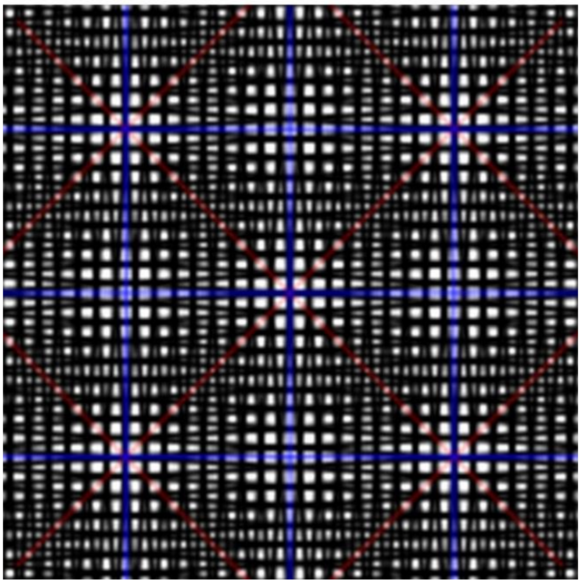

Fig S17. Moiré lattices observed in many different STEM images attesting to mono/few layered molybdenene synthesis.

6. Chemical sensing using AFM cantilever

The fundamental frequency of the rectangular cantilever beam depends on elastic modulus, mass density, length and thickness (number of layers) of the cantilever beam[S19-20].

$$f = \frac{1}{2\pi}\sqrt{\frac{E}{\rho}}\left(\frac{t}{l^2}\right) \qquad (1)$$

Where f is the fundamental resonant frequency, E is the elastic modulus, ρ is the mass density, t is thickness and l is length. If the cantilever tip will be loaded with little DNA molecules, the system can be considered equivalent to mass attached to a spring and the fundamental frequency can be given by

$$f = \frac{1}{2\pi}\sqrt{\frac{k}{m}} \qquad (2)$$

Which suggests that the square of the fundamental frequency is inversely proportional to the mass attached to the cantilever.

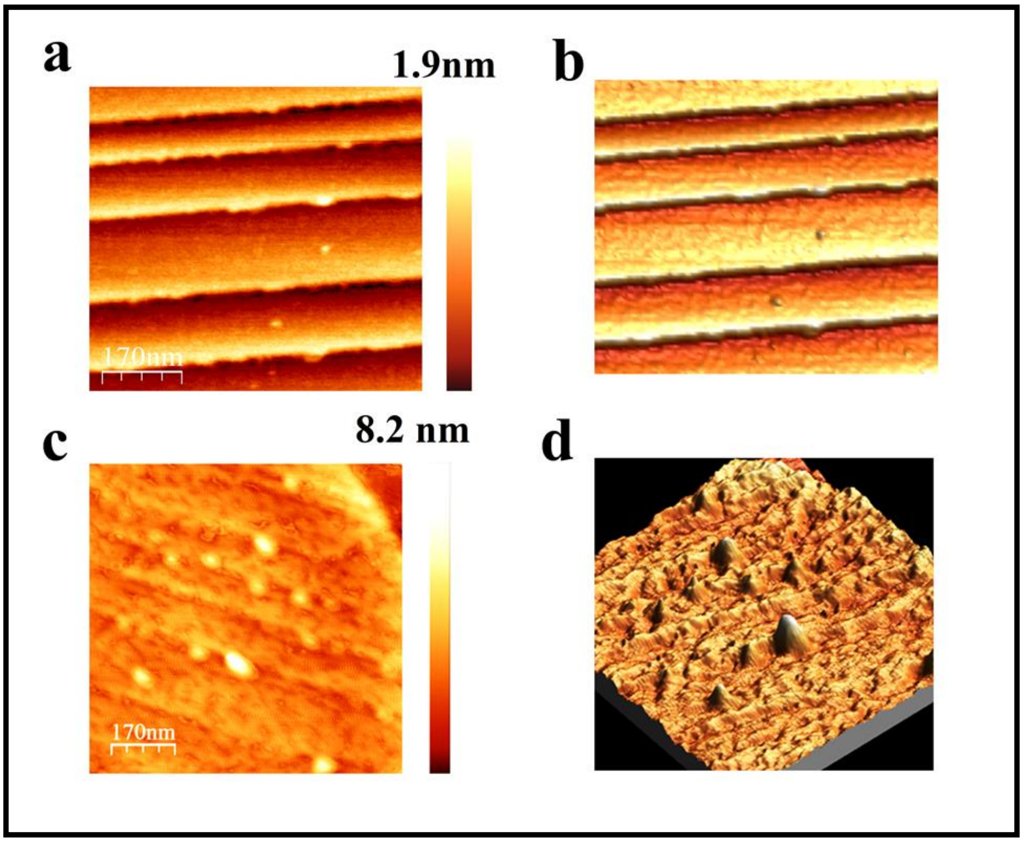

Fig S18 AFM images showing freshly prepared synthesized Mo sample in (a) 2D and (b) 3D view. Smooth surfaces are evident and staircases like features are apparent. AFM images showing Mo sample after prolonged (~24 hrs) treatment in ethanethiol in (c) 2D and (d) 3D view.

7. SERS based molecular sensing: Schematic diagram and SERS measurements sensing 10 ppm methylene blue (MB) on various anchoring surfaces are shown in Fig S19. In contrast to pure SiO_2 surface, on which there was no recognizable observed Raman peaks of MB at 10 ppm, gold thin film fairs better. However, in order to achieve distinguishable Raman fingerprints of the organic test molecules, one needs to enhance the laser power. Often, power is increased from 3 to 8 mW in the experiment and then further focusing is carried out. However, carbonization of organic molecule is one of the challenges in using gold nanoparticles or nanoparticulated thin films. Graphene, having high thermal conductivity in fact fairs better than the gold thin film. We observed that molybdenene enhances signals even at high laser power used and results for molybdenene as an anchor is comparable with that for the graphene as an anchor. We also carried out experiments with molybdenene/Au surface and in fact we could observe that noises get significantly reduced and most of the Raman fingerprints of MB could be recognized. Thus, molybdenene is strongly enhancing the Raman signal, demonstrating its application in at trace-level SERS-based molecular sensing.

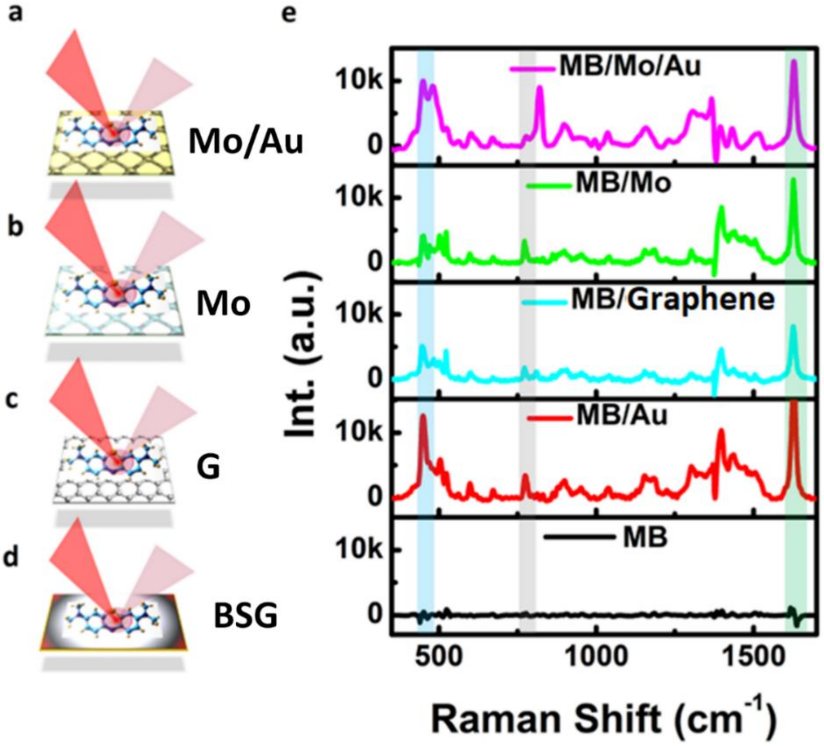

Fig S19 (a-d) The schematic representation of SERS measurement of methylene blue employing Mo transferred over gold sputtered SiO_2, Mo transferred SiO_2, graphene transferred SiO_2 and gold sputtered SiO_2 as substrates shown in Fig S19 (a), (b), (c) and (d) respectively. (e) SERS of methylene blue obtained for different substates.

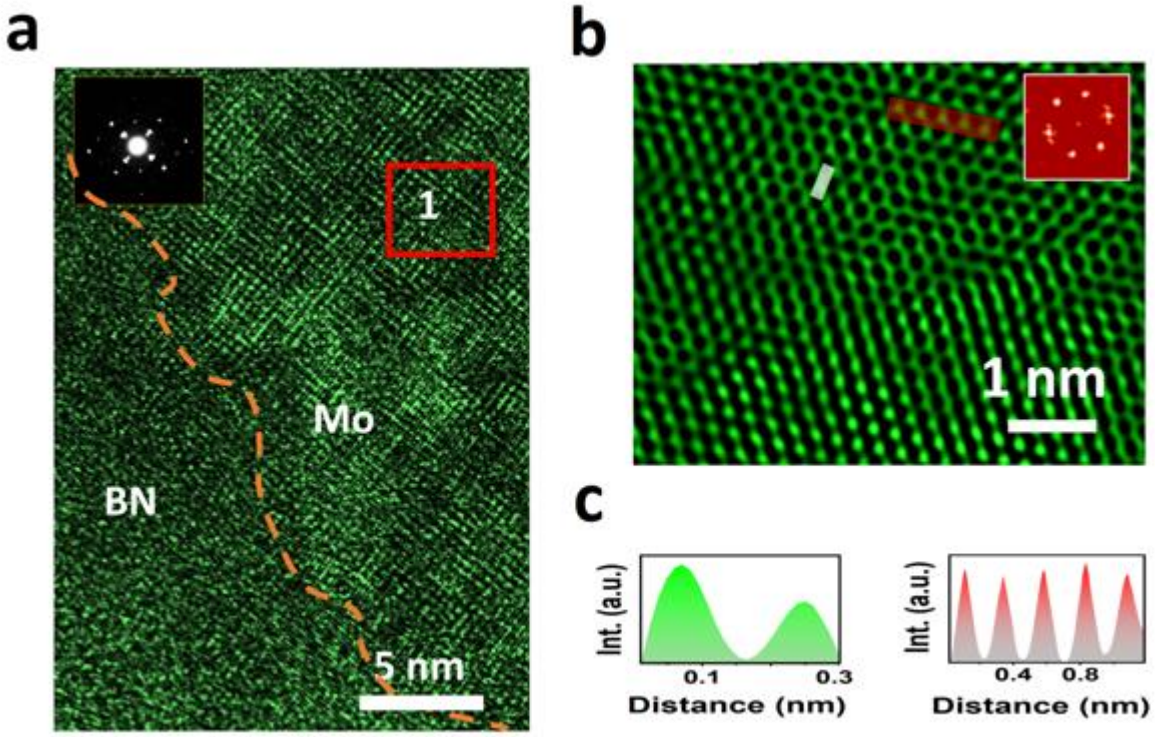

Fig S20 (a) HRTEM image at the interface of BN and Mo of Mo-BN hybrid. (b) HRTEM image of Mo-BN hybrid exhibiting hexagonal lattice symmetry. (c) Atomic line profile of image (b) taken in two directions as marked is 0.18 nm (green plot) and 0.23 nm (red plot).

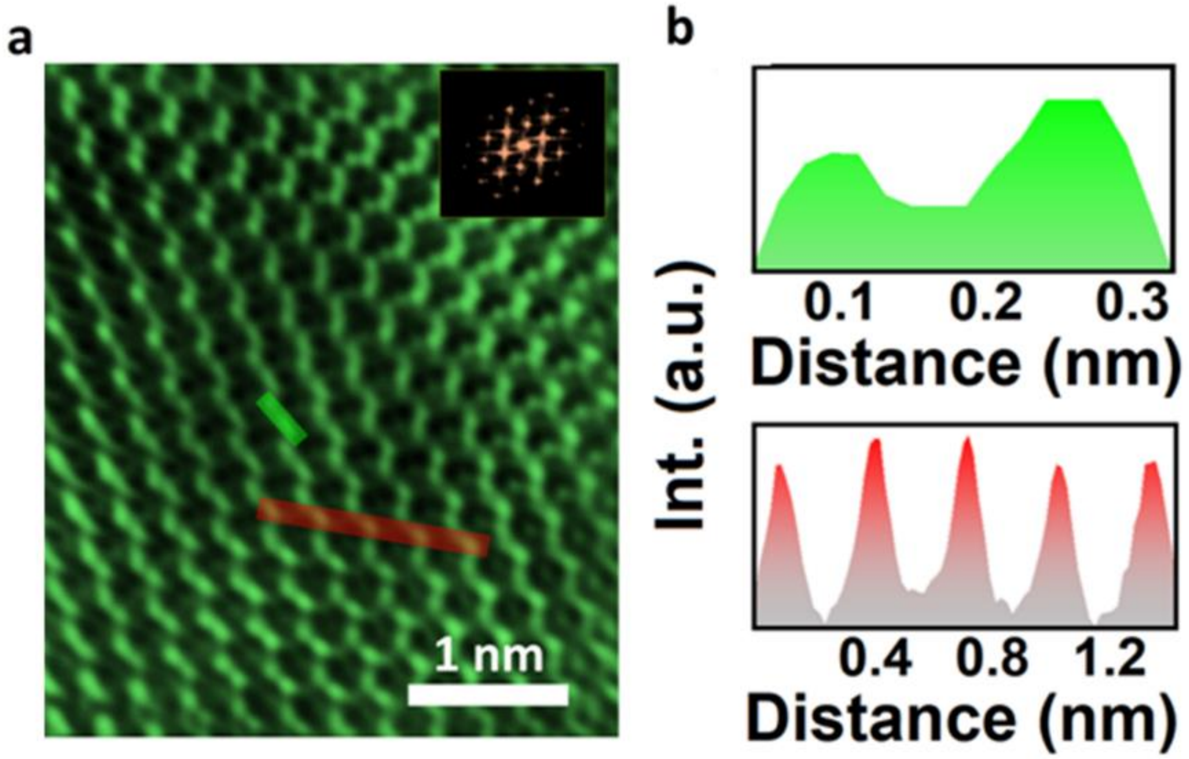

Fig S21 (a) HRTEM image of location 2 marked in Fig 5(n). (b) Atomic line profile along two directions as marked in (a) by green and red line has interatomic distance of 0.16nm (green plot and 0.32 nm (red plot).

References

S1. Thostenson, E. T. & Chou, T. W. Microwave Processing: Fundamentals and Applications. *Compos. Part A Appl. Sci. Manuf* **30**, 1055-1071 (1999).

S2. Clark, D. E., Folz, D. C. & West, J. K. Processing Materials with Microwave Energy. *Mater. Sci. Eng. A* **287**, 153-158 (2000).

S3. Zhang, X., Hayward, D. O. & Mingos, D. M. P. Dielectric Properties of MoS_2 and Pt Catalysts: Effects of Temperature and Microwave Frequency. *Catal. Letters* **84**, 225-233 (2002).

S4. Choi, W. et al. Plasma Carriers on Graphene: Relaxation of Plasma Carriers in Graphene: An Approach by Frequency-Dependent Optical Conductivity Measurement *Adv. Opt. Mater.* **6**, 1870054 (2018).

S5. Jablan, M., Buljan, H. & Soljačić, M. Plasmonics in Graphene at Infrared Frequencies. *Phys. Rev. B - Condens. Matter Mater. Phys.* (2009).

S6. Prodromakis, T. & Papavassiliou, C. Engineering the Maxwell-Wagner Polarization Effect. *Appl. Surf. Sci.* **80**, 245435 (2009).

S7. Cannon, P. Melting Point and Sublimation of Molybdenum Disulphide. *Nature* **183**, 1612-1613 (1959) .

S8. Incropera, F. P., DeWitt, D. P., Bergman, T. L. & Lavine, A. S. Fundamentals of Heat and Mass Transfer (6th edition). WILEY (2007).

S9. Bano, A., Khare, P. & Gaur, N. K. Thermal Transport Properties of Bulk and Monolayer MoS_2: An ab-initio approach. in *Journal of Physics*: Conference Series **836**, 012052 (2017).

S10. Santos, E. J. G. & Kaxiras, E. Electrically Driven Tuning of the Dielectric Constant in MoS_2 layers. *ACS Nano***7**, 10741-10746 (2013).

S11 Santos, E. J. G. & Kaxiras, E. Electric-Field Dependence of the Effective Dielectric Constant in Graphene. *Nano Lett.***13,** 898-902 (2013).

S12. Ning, M. Q. et al. Two-Dimensional Nanosheets of MoS_2: a Promising Material with High Dielectric Properties and Microwave Absorption Performance. *Nanoscale* ***7***, 15734-15740 (2015).

S13. Wang, X. et al. 2D MoS_2/Graphene Composites with Excellent Full Ku band Microwave Absorption. *RSC Adv.***6**, 106187-106193(2016).

S14. Choi et. al., Applied Surface Science **93**, 143-149 (1996).

S15. Gruenert et. al., J. Phys. Chem. **95**, 1323-1328 (1991).

S16. https://xpsdatabase.com/molybdenum-spectra-mo-metal/

S17. M. E. Davila, Sci. Rep., **6**, 20714 (2016).

S18. https://xps-database.com/molybdenum-mo-z42-molybdenum-compounds/

S19. Godin et. al., *Nanotechnology* **21**, 075501 (2010).

S20. Steffens et. al., *Sensors*, ***12***, 8278-8300 (2012).
